# *STAT1* Gain-of-Function Mutations Cause High Total STAT1 Levels With Normal Dephosphorylation

**DOI:** 10.3389/fimmu.2019.01433

**Published:** 2019-07-10

**Authors:** Ofer Zimmerman, Peter Olbrich, Alexandra F. Freeman, Lindsey B. Rosen, Gulbu Uzel, Christa S. Zerbe, Sergio D. Rosenzweig, Hye Sun Kuehn, Kevin L. Holmes, David Stephany, Li Ding, Elizabeth P. Sampaio, Amy P. Hsu, Steven M. Holland

**Affiliations:** ^1^Laboratory of Clinical Immunology and Microbiology, Immunopathogenesis Section, National Institute of Allergy and Immunology, National Institutes of Health, Bethesda, MD, United States; ^2^Sección de Infectología, Reumatología e Inmunología Pediátrica (SIRIP), Hospital Infantil Virgen del Rocío, Instituto de Biomedicina de Sevilla (IBiS), Seville, Spain; ^3^Immunology Service, Department of Laboratory Medicine, National Institutes Clinical Center, National Institutes of Health, Bethesda, MD, United States; ^4^Flow Cytometry Section, Research Technologies Branch, National Institute of Allergy and Infectious Diseases, National Institutes of Health, Bethesda, MD, United States

**Keywords:** STAT1, gain of function, dephosphorylation, protein, mRNA, monocytes, T cells, JAK inhibitors

## Abstract

Signal transducer and activator of transcription *(STAT1)1* gain of function (GOF) pathogenic variants have been associated with increased levels of phosphorylated STAT1 and STAT1-dependent cellular responses. Delayed dephosphorylation was proposed as the underlying mechanism leading to the characteristically raised pSTAT1 levels. We examined the levels of STAT1 protein and message as well as rates of STAT1 phosphorylation, dephosphorylation, and degradation associated with *STAT1* GOF pathogenic variants. Fresh peripheral blood mononuclear cells (PBMC) from 14 STAT1 GOF patients carrying 10 different pathogenic variants in the coiled-coil, DNA binding, and SH2 domains and healthy donors were used to study STAT1 levels and phosphorylation (pSTAT1) following IFNγ and IFNα stimulation. STAT1 protein levels were measured by flow cytometry and immunoblot. *STAT1* mRNA levels were measured using quantitative reverse transcription PCR. STAT1 protein degradation was studied using cycloheximide. Patient IFNγ and IFNα induced peak pSTAT1 was higher than in healthy controls. The velocity of pSTAT1 dephosphorylation after treatment of IFNγ stimulated CD14^+^ monocytes with the Janus Kinase (JAK)-inhibitor ruxolitinib was significantly faster in patient cells. STAT1 protein levels in patient CD14^+^ monocytes and CD3^+^ T cells were higher than in healthy donors. There was a strong and positive correlation between CD14^+^ STAT1 protein levels and peak pSTAT1 levels. Patient fresh PBMC *STAT1* mRNA levels were increased at rest and after 16 h of incubation. STAT1 protein degradation was similar in patient and healthy volunteer cells. Patient IFNγ receptors 1 and 2 and JAK2 levels were normal. One patient in our cohort was treated with the oral JAK inhibitor ruxolitinib. Treatment was associated with normalization of both STAT1 protein and peak pSTAT1 levels. After JAK inhibitor treatment was stopped the patient's CD14^+^ monocyte STAT1 protein and peak phosphorylation levels increased proportionally. These findings suggest that patients with *STAT1* GOF mutations have higher levels of total STAT1 protein, leading to high levels of pSTAT1 after stimulation, despite rapid STAT1 dephosphorylation and normal degradation.

## Introduction

In 2011 van de Veerdonk et al. and Liu et al., described heterozygous germline pathogenic variants in the coiled-coil domain of *STAT1* in patients with chronic mucocutaneous candidiasis (CMC) (1, 2). Soon after, mutations in the DNA binding domain were described in patients with CMC along with patients who suffered from invasive fungal infections and autoimmune phenomena (3, 4). All mutations were characterized as gain of function (GOF), due to increased STAT1-dependent cellular responses. High levels of tyrosine phosphorylated STAT1 (pSTAT1) were found in different immune cells (PBMC, CD3^+^ T cells, CD14^+^ monocytes and EBV transformed B cell lines) and cell lines (U3A and U3C) transfected with mutant vectors following stimulation with IFNγ, IFNα, IL-6, IL-21, and IL-27 (2–5). The high levels of STAT1 phosphorylation were attributed to delayed dephosphorylation, as demonstrated by elevated pSTAT1 levels up to 120 min from stimulation and by use of the kinase inhibitor staurosporine (2–5). Most of these data were generated by immunoblotting. We revisited these high pSTAT1 levels, along with the kinetics of STAT1 phosphorylation and dephosphorylation, *STAT1* message level, STAT1 protein level and degradation, in primary cells from patients carrying *STAT1* GOF pathogenic variants. We also evaluated the effect of oral JAK inhibitor treatment with ruxolitinib on STAT1 phosphorylation and protein levels.

## Materials and Methods

### Patients, Controls, and PBMC Isolation

Fourteen patients carrying *STAT1* GOF mutations were enrolled (2014–2017) on approved NIH protocols and provided written informed consent. Healthy donor blood samples were obtained under approved protocols through the Department of Transfusion Medicine, Clinical Center, NIH.

Patient and healthy donor peripheral blood mononuclear cells (PBMC) were isolated by density-gradient centrifugation using lymphocyte separation media (Lonza). PBMC were re-suspended in RPMI culture media (Gibco), supplemented with pyruvate (100 mM, Sigma Aldrich), glutamate (200 mM, Life Technologies), penicillin/streptomycin (100 U/100 μg/ml, Life Technologies), 10% fetal bovine serum (Serum Source International), and 20 mM HEPES (GE).

### PBMC Stimulation

PBMC were stimulated in polystyrene round-bottom tubes (Becton Dickinson Falcon), at 10^7^/ml in 100 mcl total volume. Stimulation was performed with IFNγ 1b (ACTIMMUNE) 400 or 800 U/ml, or IFNα (PBL) 100 ng/ml.

### Intracellular Staining for STAT1, pSTAT1, STAT2, and JAK2

Intracellular pSTAT1, total STAT1, total STAT2, total JAK2, and STAT1 dephosphorylation kinetics were determined by FACS analysis. Freshly isolated PBMC were re-suspended at 10^6^/100 mcl in plain RPMI and serum starved for 30 min. Cells were incubated with anti-human CD14-FITC or anti-human CD14-APC (Becton Dickinson 555397 and 555399, respectively) and anti-human CD3 APC-eFluor® 780 (eBioscence 47-0037-42) or anti-human CD3 APC-H7 (Becton Dickinson 560275). Cells were stimulated with IFNγ 400 or 800 U/ml for 15–180 min at 37°C, or with IFNα 100 ng/ml for 30 min, fixed with PFA 2% at 37°C for 10 min, permeabilized with 100% methanol on ice for 30 min, washed with PBS/2%FBS, and incubated for 1 h in the dark at 4°C with combinations or one of the following antibodies: anti-human pSTAT1-Alexa fluor 647/ PerCP-Cy™5.5 (Y701) (Becton Dickinson 612597 and 560113, respectively), anti-human STAT1 N-terminus-Alexa Fluor® 647/PE (Becton Dickinson 558560 and 558537, respectively), anti-human STAT2 (Cell Signaling 72604) or anti-human JAK2-PE (Cell Signaling 5941 s) mixed in Fix and Perm Permeabilization Medium (Medium B) (Life technologies). Antibodies with the same fluorochrome (e.g., anti-human pSTAT1 AF647, anti-human STAT1 AF647, or anti-human STAT1 PE, anti-human JAK2 PE) were never used in the same tube. Alexa Fluor® 647 Mouse IgG1 κ Isotype control (Becton Dickinson 557783) and rabbit (DA1E) mAb IgG XP® Isotype Control (Cell Signaling 3900) were used as a control for anti-human STAT1 AF647 (Becton Dickinson) and anti-human STAT2 (Cell Signaling) primary antibodies, respectively. For STAT2, cells were washed with PBS/2%FBS and incubated with anti-Rabbit IgG Alexa Fluor® 488 Conjugated antibody (Cell Signaling 4412) for 30 min. Before analysis each sample was washed once with PBS/2%FBS and resuspended in PFA 1%. All data were collected with FACSCalibur™, LSRFortessa™ or LSR II (all Becton Dickinson) and analyzed with FlowJo software (Treestar, Ashland, OR, USA).

### Kinase Inhibitor Use in STAT1 Dephosphorylation Assays

To study STAT1 dephosphorylation we sought a potent kinase inhibitor and optimal concentrations for complete inhibition of STAT1 phosphorylation. We compared the kinase inhibitor staurosporine and the JAK inhibitor ruxolitinib for their effects on healthy donor CD14^+^ STAT1 phosphorylation after IFNγ stimulation. Healthy donor PBMC were incubated with anti-human CD14-FITC (Becton Dickinson 555397) for 15 min. Either staurosporine (both products from Sigma Aldrich and from Selleckchem) or ruxolitinib (Selleckchem) were added at concentrations of 25, 50, 100, 200, 500, or 1,000 nM for 15 min, following which cells were stimulated with IFNγ (ACTIMMUNE) 800 U/ml for 15 min at 37°C. After 15 min of stimulation cells were fixed with PFA 2% for 10 min at 37°C. Cells were stained for pSTAT1 as described above.

To determine the kinetics of dephosphorylation in healthy controls, fresh PBMC were incubated with anti-human CD14-FITC as above, and then stimulated with IFNγ 800 U/ml at 37°C. Fifteen minutes after IFNγ stimulation staurosporine at final concentration of 500 nM, or 1,000 nM or ruxolitinib at 1,000 nM were added to the media. Cells were incubated for 15–90 min after the administration of staurosporine or ruxolitinib, and then fixed and stained for pSTAT1 as described above. Finally, to understand patient dephosphorylation kinetics, both fresh patient and fresh healthy control PBMC were incubated with anti-human CD14-FITC for 15 min, and then stimulated with IFNγ 800 U/ml, at 37°C. Fifteen minutes after stimulation ruxolitinib at a final concentration of 1,000 nM was added. Cells were incubated at 37°C for 15–120 min after the administration of ruxolitinib, and then fixed and stained for pSTAT1 as described above.

### STAT1 Degradation Assay

We studied STAT1 degradation using the protein synthesis inhibitor cycloheximide (Sigma Aldrich). 10^6^ patient or healthy control fresh PBMC in 200 mcl of culture media (see above) were incubated with cycloheximide at 100 ng/ml for 4 and 16 h. Each patient and healthy volunteer had STAT1 protein levels determined at 0, 4, and 16 h of incubation in culture with and without cycloheximide. Three and a half and 15.5 h after incubation, cells were live/dead stained (LIVE/DEAD™ Fixable Aqua Dead Cell Stain Kit, Thermo Fisher) for 10 min, incubated with anti-human CD14 FITC conjugated antibody (BD) for 15 min, washed with 37°C RPMI media and fixed with PFA 2% for 10 min at 37°C. Cells were than permeabilized with methanol 3 ml at −20°C in the dark. All samples were incubated at the same time with anti-human STAT1 Alexa Fluor® 647 (Becton Dickinson 558560), anti-human CD3 APC-H7 (Becton Dickinson 560275), anti-huamn CD11b Alexa Fluor® 700 (Becton Dickinson 557918) and anti-human CD64 PE-Cy™7 (Becton Dickinson 561191) antibodies. After 1 h of incubation cells were washed as described above. All data were collected with LSR II (Becton Dickinson) and analyzed with FlowJo software (Treestar, Ashland, OR, USA).

### Extracellular Staining for IFNγ Receptors 1 and 2

Levels of IFNγ receptors 1 and 2 were determined by FACS analysis. Freshly isolated PBMC were resuspended at 10^6^/100 mcl as described above. Cells were incubated for 30 min in the dark at 4°C with anti-human CD14-FITC (Becton Dickinson 555397), and anti-human CD119-PE (IFNγR1, Becton Dickinson 558937) or anti-human IFNγR2-APC (R&D FAB773A), washed with PBS/2%FBS and fixed with PFA 1%. Mouse anti-IgG2b, κ-PE (Becton Dickinson 555058) and Goat anti-IgG-APC (R&D IC108A) were used as isotype controls. Data were collected with LSRFortessa™ (Becton Dickinson) and analyzed with FlowJo software (Treestar, Ashland, OR, USA).

### Immunoblotting Assays

Immunoblotting was used to determine STAT1 protein and pSTAT1 levels in both patient and healthy donor PBMC, at rest and after 30 min of IFNγ stimulation.

To optimize immunoblotting for quantitation, we determined the linear range for STAT1 and beta actin antibodies that were used (6, 7). Both STAT1 and beta actin had low and narrow linear ranges between 2 and 16 mcg total protein (Figure S6). Hence, we loaded only 10–15 mcg total protein per sample.

Data were acquired using ChemiDoc MP imaging system (Bio Rad) and analyzed using Image Lab software (Bio-Rad Laboratories; version 5.2.1). Please refer to the online Supplementary Material for complete details.

### Quantitative Reverse Transcription Polymerase Chain Reaction (RT-qPCR) Analysis

Fresh PBMC relative *STAT1* mRNA levels were determined by qPCR.

Patient and healthy volunteer PBMC were stimulated with IFNγ 400 IU/ml for 16 h. For each patient and healthy volunteer, a non-stimulated control was prepared, which was run in parallel for 16 h. Patient and healthy volunteer baseline controls were obtained from fresh PBMC, immediately after their separation from whole blood. Cells were spun down at 4° degrees, and then washed with cold PBS. Total RNA was extracted using RNeasy mini kit (QIAGEN) with a DNase reaction (QIAGEN). cDNA was generated using High-Capacity cDNA Reverse Transcription (Applied Biosystems) and oligo dT priming. Relative *STAT1* mRNA levels were determined with Taqman probes (Life, Hs01013996), using the ddCt algorithm. The results were normalized with respect to the values obtained for the endogenous Beta Actin (Life, Hs99999903) cDNA. All procedures were performed with technical triplicates and with biological duplicates or triplicates when available.

### STAT1 Sequencing

Genomic DNA was extracted from whole blood, amplified and sequenced for *STAT1* exons and flanking splice sites as previously described (3).

### Statistics

Data from all experiments acquired on the same flow cytometer machine, using the same settings and the same fluorochromes were analyzed in raw values of geometric mean of fluorescence. Data were also analyzed as average of the same day healthy donors pSTAT1 and STAT1 protein levels as measured by geometric mean of fluorescence. Each healthy donor and patient pSTAT1 or STAT1 level was expressed as percentage of the same day healthy donors' average level.

Statistical analyses were performed using GraphPad Prism7 (La Jolla, CA, USA). Results are expressed as mean ± standard deviation (SD) unless otherwise indicated. For group comparisons, the parametric independent Student's *t*-test was used to analyze differences in continuous variables. The Shapiro-Wilk normality test was used to verify Gaussian distribution. Comparison between groups that did not pass the normality test was performed using the Mann-Whitney non-parametric test, for continuous variables.

Simple linear regression was performed to analyze changes in pSTAT1 levels over time. To test whether dephosphorylation kinetics differed between controls and patients we used a method equivalent to analysis of covariance (ANCOVA) (8). The Pearson product-moment correlation coefficient was used for analysis of correlations between variables. The *p*-value for significance was set at <0.05.

## Results

**Figure 1:**
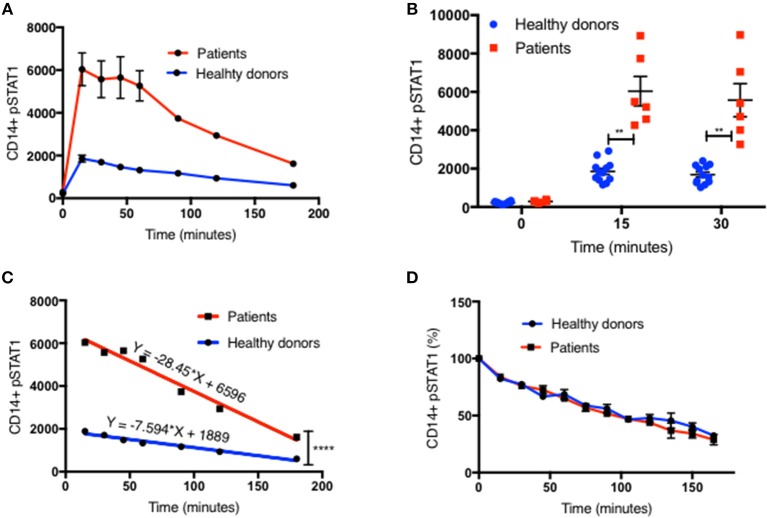
Increased peak pSTAT1 levels with normal STAT1 dephosphorylation rate in GOF CD14^+^ monocytes. **(A)** Average CD14^+^ monocytes pSTAT1 level at rest (time 0) and up to 3 h of IFNγ stimulation in GOF patients (red line, *n* = 6) and healthy donors (blue line, *n* = 12), as measured by flow cytometry with an anti pSTAT1 AF647 antibody. Levels are expressed in geometric mean of fluorescence. **(B)** Patients' (red squares, *n* = 6) and healthy donors' (blue dots, *n* = 12) pSTAT1 at rest and after 15′ and 30′ of IFNγ stimulation, as measured by flow cytometry. Each red square represents the average of repeated measurement (1–3) of each patient. Each blue dot represents one measurement of one healthy control. Comparisons between the two groups were performed for each time point independently. **(C)** Linear regression lines of pSTAT1 level over time (minutes), from peak level, starting 15 min after IFNγ stimulation, of patients' (red lines, *n* = 6) and healthy controls' (blue lines, *n* = 12) CD14^+^ monocytes, as measured by flow cytometry. **(D)** Average CD14^+^ monocytes pSTAT1 level over time as expressed in percentage from peak level of each tested healthy donor (blue line, *n* = 36) or GOF patient (red line, *n* = 12). Peak phosphorylation point was defined as time zero for each patient or healthy control, independently. **P < 0.01; ****P < 0.0001, by *t*-test **(B)** or ANCOVA **(C)**. Quantitative data represent mean ± SEM.

We examined pSTAT1 (Tyr701) levels in CD14^+^ monocytes of 13 patients with 10 different coiled-coil, DNA binding, and SH2 domains *STAT1* pathogenic variants by flow cytometry and compared them to healthy controls. Patients' clinical data were collected from medical records and are briefly summarized in Table 1. pSTAT1 levels were measured at rest and with IFNγ stimulation. We focused first on IFNγ since it potently induces STAT1 phosphorylation and homo-dimerization (14, 15). We focused on CD14^+^ monocytes because of their relatively high levels of IFNγ receptors 1 and 2, which allow for rapid activation of STAT1 (16). In 25 separate experiments, patient cells were compared with one or two healthy controls per experiment, such that each patient was compared to 2–5 separate healthy controls. We looked at the kinetics of pSTAT1 formation in CD14^+^ monocytes stimulated with IFNγ in healthy controls and GOF patients (up to 180 min) (Figure 1A and Figure S1A). In both healthy control and patient cells, pSTAT1 levels peaked around 15 to 30 min and gradually decreased toward baseline. However, they typically did not reach pre-stimulation baseline levels, even after 3 h, in either healthy control or patient cells.

**Table 1:** Genetics and main phenotypical features of 14 patients with STAT1 GOF mutations.

	**Mutation (cDNA)/(amino acid)**	**Affected domain**	**Sex**	**Age (y)**	**Clinical onset**	**CMC**	**Infections**	**Autoimmunity/inflammatory**	**Endocrinopathy**	**Immunosuppressive therapy at the time of the study**
**P1**	c.(493G>C) D165H (2)	CCD	M	30	1st year of life	Oral cavity, esophagus, skin and genital mucosa	Recurrent sinopulmonary infections; HSV esophagitis; recurrent herpes zoster	Upper GI ulcers	Type 1 DM	None
**P2**	c.(704A>G) E235G (9)	CCD	F	62	1st year of life	Oral cavity, skin and genital mucosa	Recurrent sinopulmonary infections; bacterial skin infections with abscesses; recurrent HSV labialis	Alopecia		None
**P3**	c.(704A>G) E235G (9)	CCD	F	34	1st week of life	Oral cavity	Recurrent sinopulmonary infections			None
**P4**	c.(800 C>T) A267V (1, 3)	CCD	M	19	6 months old	Oral cavity, esophagus and skin	Recurrent RSV; *Mycobacterium fortuitum* lymphadenitis			None
**P5**	c.(820C>T) R274W (1, 10)	CCD	F	30	1st year of life	Oral cavity, esophagus and genital mucosa	Recurrent pneumonia; recurrent bacteremia; bacterial skin infections	Myopathy; SLE	Type I DM	Ruxolitinib
**P6**	c.(821G>A) R274Q (1, 2, 10)	CCD	F	30	3 years old	Oral cavity, esophagus, skin and genital mucosa	Recurrent sinopulmonary infections; bacterial skin infections with abscesses; recurrent herpes zoster			None
**P7**	c.(821G>A) R274Q (1, 2, 10)	CCD	M	4	3 years old	Oral cavity and genital mucosa				None
**P8**	c.(963A>T) R321S (11, 12)	DBD	F	18	6 years old	Skin	Recurrent sinopulmonary infections; bacterial skin infections; dermatophytosis; recurrent herpes zoster	Alopecia	Hypothyroidism; GH deficiency	None
**P9**	c.(963A>T) R321S (11, 12)	DBD	F	21	6 years old	Oral cavity and skin	Recurrent sinopulmonary infections; bacterial skin infections with abscesses; dermatophytosis; warts	Autoimmune hepatitis; alopecia	Type I DM	None
**P10**	c.(963A>T) R321S (11, 12)	DBD	M	25	1st year of life	Oral cavity, esophagus and skin	Recurrent sinopulmonary infections; disseminated MAC; severe acute varicella zoster infection; Parvo B19 viremia; BK viruria; HCV	Cytopenia; HLH	GH deficiency	None
**P11**	c.(983A>G) H328R	DBD	M	10	1st year of life		Recurrent sinopulmonary infections	Colitis	Hypothyroidism; Type I DM; GH deficiency	HSCT with low chimerism 5 years prior to the study
**P12**	c.(1057G>A) E353K (3)	DBD	M	27	14 years old		Disseminated *coccidioidomycosis*; dermatophytosis			Ruxolitinib
**P13**	c.(1154C>T) T385M (3, 4, 10)	DBD	M	27	1st week of life	Oral cavity and skin	Histoplasma pneumonia; recurrent herpes zoster; *Mycobacterium fortuitum* lymphadenitis			None
**P14**	c.1885C>T H629Y (13)	SH2D	F	25	1st year of life	Oral cavity and genital mucosa	Recurrent pneumonia requiring lobectomy; rectal abscesses; *C. dif*; recurrent herpes zoster	Rectovaginal fistula	Hypothyroidism	None

*CCD, coiled-coil domain; CMC, chronic mucocutaneous candidiasis; C. diff., Clostridium difficile; DBD, DNA-binding domain; DM, Diabetes mellitus; GH, growth hormone; HSCT, Hematopoietic stem cell transplantation; HCV, Hepatitis C virus; HSV, Herpes simplex virus; MAC, Mycobacterium avium complex; RSV- respiratory syncytial virus*.

*References for the nine mutations that were published in the past are in parentheses*.

Patient CD14^+^ monocyte pSTAT1 levels at rest were not significantly different from healthy control cells after 30 min of serum starvation (Figure 1B and Figures S1B, S2A,B, S3A1). With IFNγ stimulation patient cell peak pSTAT1 levels were significantly higher than those of healthy volunteers (Figure 1B and Figures S1B, S2A,B, S3A1). The mean pSTAT1 levels of 13 patients tested compared to 40 healthy donors was 2.7 times higher (±0.24) after 15 min and 2.8 times higher (±0.25) after 30 min of IFNγ stimulation, respectively (*P* < 0.0001 for all) (Figure S1B).

The pattern of post-peak decline in pSTAT1 was linear in both patient and healthy volunteer cells (Figures 1A,C and Figures S1A,E). However, the slope of pSTAT1 decrease was 3.1 times steeper on average in GOF patient cells than healthy controls (Figure 1C and Figure S1E). We analyzed the same raw flow cytometry data looking at the average absolute decrease in pSTAT1 level per minute, after it reached its peak level 15–30 min after IFNγ stimulation. The average delta pSTAT1 per minute was higher in the patient group at every tested time point, however these differences were significant during the first 15 min after pSTAT1 reached its peak level, in one set of experiments (Figure S1C) and during the first 30–60 min after pSTAT1 reached its peak in a second set of experiments (Figure S1D).

We looked at the rate of decrease in pSTAT1 level as a percentage of its peak level in cells from healthy donors and patients (Figure 1D). For each healthy donor and patient, pSTAT1 levels were expressed as % of maximum. The peak pSTAT1 level as measured by flow cytometry and expressed in geometric mean fluorescence level was defined as 100%, and the peak pSTAT1 time point was defined as time 0. As can be seen in Figure 1D, when expressed as % of maximum level, the average decrease in pSTAT1 level over time in both healthy volunteer and GOF patient cells was almost identical. As seen in Figure S1F the decreases in pSTAT1 levels were linear and superimposable when the values were expressed in percentages. The equations of the regression lines were almost identical for both healthy controls and GOF patients. Therefore, the rate of decrease in pSTAT1 from its peak level is similar in GOF patients and healthy controls.

**Figure 2:**
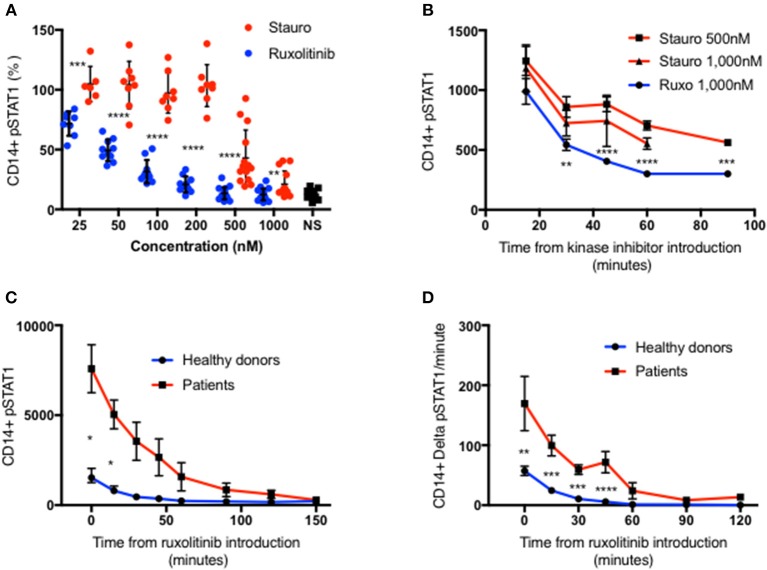
Ruxolitinib is more potent than staurosporine in inhibiting STAT1 phosphorylation. **(A)** Healthy donors CD14^+^ monocytes pSTAT1 level after 15′ of IFNγ stimulation with pre-incubation with a kinase inhibitor ruxolitinib (blue dots, *n* = 16) or staurosporine (red dot, *n* = 14) at increasing concentration (25–1,000 nM). Each individual's pSTAT1 level is expressed in percentage of the same healthy donor pSTAT1 level after 15′ of IFNγ stimulation, without pre-incubation with a kinase inhibitor. **(B)** CD14^+^ monocytes pSTAT1 level 15–90′ after introduction of staurosporine (500 or 1,000 nM) or ruxolitinib (1,000 nM) to healthy donors fresh PBMC stimulated with IFNγ for 15 min. Levels are expressed in geometric mean of fluorescence. **(C)** Average CD14^+^ monocytes pSTAT1 level of healthy controls (blue, *n* = 9) and patients (red, *n* = 3 with 1–3 repeated measurements per patient) over 2.5 h after introduction of ruxolitinib 1,000 nM to fresh PBMC, stimulated first with IFNγ for 15 min. Levels are expressed in geometric mean of fluorescence. **(D)** Average decrease per minute in CD14^+^ monocytes pSTAT1 level in healthy controls (blue, *n* = 9) and patients (red, *n* = 3) over 2 h after introduction of ruxolitinib 1,000 nM to fresh PBMC, stimulated first with IFNγ for 15 min. *P < 0.05; **P < 0.01; ***P < 0.001; ****P < 0.0001, by *t*-test. Quantitative data represent mean ± SEM.

To explain these observations, we sought to examine STAT1 dephosphorylation following complete blockade of STAT1 phosphorylation after peak pSTAT1. We examined the relative efficacies of staurosporine and ruxolitinib in blocking STAT1 phosphorylation in healthy donor CD14^+^ monocytes. Ruxolitinib blocked STAT1 phosphorylation better than staurosporine at every concentration tested (Figure 2A). Moreover, staurosporine, a non-specific kinase inhibitor, had paradoxical activity at lower concentrations in some healthy controls (Figure 2A). Ruxolitinib 500 or 1,000 nM completely inhibited STAT1 phosphorylation following IFNγ stimulation, blocking IFNγ signaling. Neither 500 nor 1,000 nM of staurosporine completely blocked IFNγ signaling (Figure 2A), regardless of the commercial source of staurosporine. We compared the velocity of pSTAT1 dephosphorylation in cells treated with 500 nM of staurosporine, 1,000 nM of staurosporine or 1,000 nM of ruxolitinib 15 min after stimulation with IFNγ. The average pSTAT1 level was lower at every tested time point in the cells treated with ruxolitinib compared to cells treated with staurosporine (Figure 2B).

In light of these findings we chose to use ruxolitinib 1,0000 nM to study pSTAT1 dephosphorylation. We stimulated fresh PBMC from three patients with three different GOF pathogenic variants and nine healthy controls with IFNγ. At 15 min after stimulation we added ruxolitinib 1,000 nM and monitored pSTAT1 over time by flow cytometry. Average patient CD14^+^ monocyte pSTAT1 level 15 min after IFNγ stimulation was 4.5 ± 0.79 times the average control level (*p* < 0.05) (Figure 2C). Healthy volunteer average pSTAT1 levels returned to baseline by 60 min after ruxolitinib, while patient average pSTAT1 levels came back to baseline after ruxolitinib only by 150 min (Figure 2C). As seen in Figure 2D the absolute decrease in STAT1 phosphorylation per minute was significantly higher in the patient group at every time point during the first hour after ruxolitinib administration. Therefore, more pSTAT1 was being dephosphorylated per minute in GOF patients.

These data indicated that the absolute decrease in the level of pSTAT1 molecules was significantly greater in the GOF patient group, while the rate of GOF pSTAT1 dephosphorylation was equivalent to normal. However, it was also clear that pSTAT1 levels in STAT1 GOF patients were elevated. Therefore, we sought explanations for the persistence of high levels of pSTAT1 in STAT1 GOF patient cells. We hypothesized that increased levels of total STAT1 might explain the increased levels of pSTAT1.

**Figure 3:**
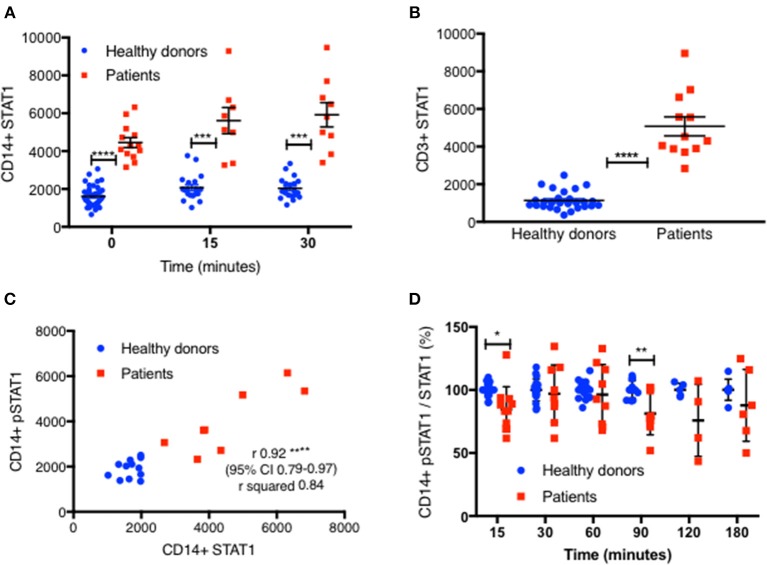
STAT1 protein levels are increased in GOF CD14^+^ monocytes and CD3^+^ lymphocytes. **(A)** GOF patients' (red squares, *n* = 13) and healthy controls' (blue dots, *n* = 38) CD14^+^ monocytes STAT1 protein level, at rest, 15′ and 30′ after IFNγ stimulation, as measured by flow cytometry with anti-STAT1 AF647 antibody. Each red dot represents the average of repeated measurement of one patient (1–3). Each blue dot represents one measurement of a healthy control. Comparisons between the two groups were performed for each time point independently. Levels are expressed in geometric mean of fluorescence. **(B)** CD3^+^ cells STAT1 protein level in 12 tested GOF (red) patients compared with healthy controls (blue, *n* = 27), as measured by flow cytometry. Each red dot represents the average of repeated measurement of one patient (1–4). Each blue dot represents one measurement of a healthy control. **(C)** Pearson correlation of STAT1 protein level (x axis) vs. peak pSTAT1 level (y axis) in CD14^+^ monocytes of both patients (red squares, *n* = 8) and healthy controls (blue circles, *n* = 12) as measured by flow cytometry. Levels are presented in geometric mean of fluorescence. **(D)** CD14^+^ monocytes pSTAT1 level corrected by STAT1 protein level in healthy controls (blue dots, *n* = 5–20 per time point) and GOF patients (red squares, *n* = 4–11 per time point) 15–180′ after IFNγ stimulation. Data is presented in percentages of healthy controls average level. *P < 0.05; **P < 0.01; ***P < 0.001; ****P < 0.0001, by t test **(A,B,D)**, and Pearson correlation **(C)**. Quantitative data represent mean ± SEM.

We measured total STAT1 protein levels in CD14^+^ and CD3^+^ cells by flow cytometry in 14 patients with 10 different mutations (Table 1), in 22 experiments alongside 44 healthy controls. Each patient was compared with 1–6 healthy controls. All 14 patients had increased total STAT1 protein levels in CD14^+^ monocytes at rest and with IFNγ stimulation (Figure 3A and Figures S3A2, S4A). Patient CD14^+^ monocyte mean STAT1 protein levels at rest were 2.9 ± 0.34 times healthy controls (*P* < 0.0001) (Figure S4A). After 15 and 30 min of IFNγ stimulation patient mean CD14^+^ monocyte STAT1 protein levels were 2.9 ± 0.46 and 3.1 ± 0.38 times healthy donor average STAT1 protein levels, respectively (*p* = 0.004 and *p* = 0.0005, respectively). CD3^+^ cell STAT1 protein levels were also increased in the 12 tested patients. The mean patient CD3^+^ STAT1 level was 4.5 ± 0.44 times the average healthy donor levels (*P* < 0.0001) (Figure 3B and Figure S3A2).

We next looked to see whether there was a correlation between total CD14^+^ monocytes STAT1 protein levels and stimulated peak pSTAT1 levels. The Pearson r coefficient of CD14^+^ monocytes total STAT1 protein vs. peak pSTAT1 level as measured by flow cytometry was 0.92 (95% CI 0.79–0.97, *R*^2^ 0.84; *p* < 0.0001) (Figure 3C). Therefore, the higher level of total STAT1 protein was directly correlated with higher peak pSTAT1 levels. When we corrected patient and healthy control CD14^+^ monocytes pSTAT1 levels for their total STAT1 levels we found that the average ratio of pSTAT1 to total STAT1 protein in patient cells after IFNγ stimulation was the same or slightly lower than that of healthy controls (Figure 3D).

**Figure 4:**
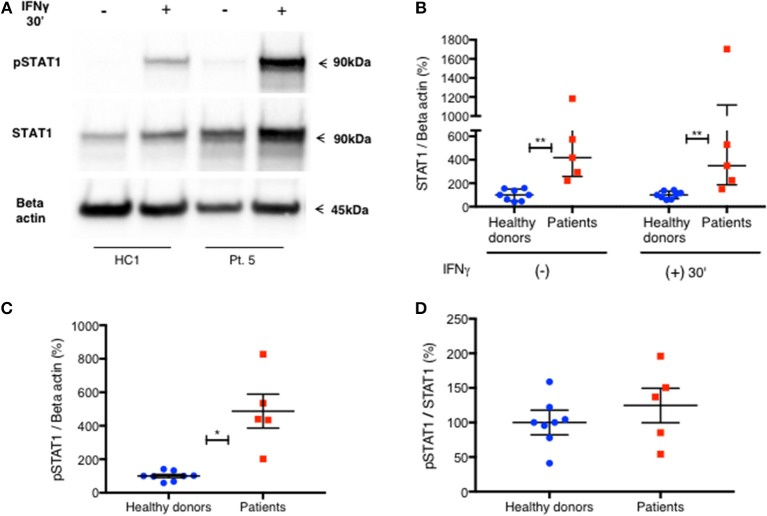
Increased level of PBMC STAT1 protein and pSTAT1 in GOF patients—by immunoblotting. **(A)** Pt. 5 pSTAT1 and STAT1 protein levels at rest and after 30′ of IFNγ stimulation, compared with a healthy control, as measured by immunoblotting (blots of STAT1 and Beta Actin are from the same gel. pSTAT1 blots are from a duplicate gel of the same samples). **(B)** STAT1 protein/Beta actin ratio at rest and after 30′ of IFNγ stimulation in five patients (red squares) compared with eight healthy controls (blue dots), as measured by immunoblotting. Data is presented in percentages of the same day healthy controls' average ratio of STAT1/Beta actin, as measured by optical densitometry (OD). **(C)** pSTAT1/Beta actin ratio after 30′ of IFNγ stimulation in five patients (red squares) compared to eight healthy controls (blue dots), as measured by immunoblotting. Data is presented in percentages of the same day healthy controls' average ratio of pSTAT1/Beta actin, as measured by optical densitometry (OD). **(D)** pSTAT1/STAT1 protein ratio, 30 min after IFNγ stimulation level in healthy controls (blue dots, *n* = 8) and GOF patients (red squares, *n* = 5) as measured by immunoblotting. Data is presented in percentages of the same day healthy controls' pSTAT1/STAT1average ratio as measured by optical densitometry (OD). *P < 0.05; **P < 0.01, by Mann-Whitney **(B)** or t test **(C,D)**. Quantitative data represent median with interquartile range **(B)** or mean ± SEM **(C,D)**.

To confirm these findings, we used immunoblotting to measure total STAT1 and pSTAT1 levels in lysates of PBMC stimulated with IFNγ. The relative levels of total STAT1 and pSTAT1 protein compared to beta actin levels were significantly higher in patients than healthy volunteers (Figures 4A–C). By optical densitometry (OD), patient median relative total STAT1 protein levels at rest, and 30 min after IFNγ stimulation were 4.2 and 3.5 times those of healthy volunteers, respectively (Figure 4B, *p* = 0.002 for all), while patient relative pSTAT1 levels 30 min after IFNγ stimulation were 4.9 times healthy volunteer levels (Figure 4C, 487%±101 vs. 100%±10, *p* = 0.019). After correcting pSTAT1 levels for total STAT1 levels, there was no significant difference between patient and control pSTAT1 levels (Figure 4D). Three of the five tested patients had increased levels of pSTAT1 at baseline (Supplementary Material), but these levels were below the level of reliable quantification (0.5–3% of the OD of IFNγ stimulated samples). We did not find this by flow cytometry based pSTAT1 assay, likely because of the serum starvation used for flow cytometry.

**Figure 5:**
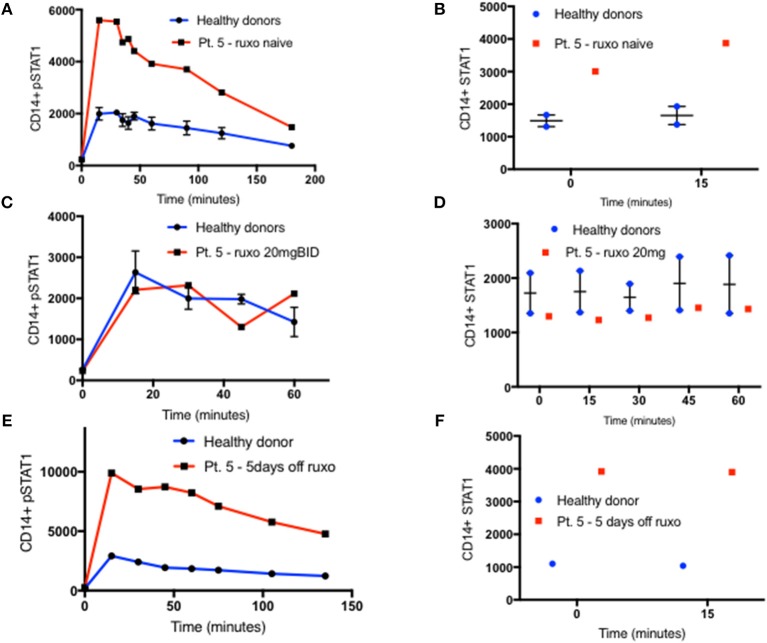
STAT1 and pSTAT1 normalization with oral ruxolitinib treatment. **(A)** Patient five (red line) and two healthy controls (blue line) CD14^+^ monocytes pSTAT1 level 0–180′ after IFN**γ** stimulation, as measured by flow cytometry while the patient was ruxolitinib naïve. Levels are expressed in geometric mean of fluorescence. **(B)** Patient five (red squares) and two healthy controls (blue dots) CD14^+^ monocytes STAT1 protein level at rest and 15 min after IFN**γ** stimulation, as measured by flow cytometry while the patient was ruxolitinib naïve. **(C)** Patient five (red line) and two healthy controls (blue line), CD14^+^ monocytes pSTAT1 level 0–60′ after IFN**γ** stimulation while the patient was on oral ruxolitinib 20 mg BID. **(D)** Patient five (red squares) and two healthy controls (blue dots) CD14^+^ monocytes STAT1 protein level at rest and up to 60 min after IFN**γ** stimulation while the patient was on oral ruxolitinib 20 mg BID. **(E)** Patient five (red line) and a healthy control (blue line) CD14^+^ monocytes pSTAT1 protein level 0–135′ min after IFN**γ** stimulation, 5 days after the patient stopped ruxolitinib treatment. **(F)** Patient five (red squares) and a healthy control (blue dots) CD14^+^ monocytes STAT1 protein level at rest and 15 min after IFN**γ** stimulation, 5 days after the patient stopped ruxolitinib treatment. Quantitative data represent mean ± SEM.

Oral ruxolitinib has been reported to normalize STAT1 phosphorylation in patients with STAT1 GOF mutations (17). Therefore, we hypothesized that the normalization of STAT1 phosphorylation might be associated with normalization of total STAT1 protein levels. Patient five was started on ruxolitinib treatment for CMC and arthritis. Prior to treatment, both her CD14^+^ monocyte STAT1 protein and CD14^+^ monocyte peak pSTAT1 levels were 2.3 and 2.6 times those of healthy volunteers, respectively (Figures 5A,B). After 14 days of oral ruxolitinib treatment, both her total STAT1 protein and phosphorylated STAT1 were similar to healthy volunteer levels (Figures 5C,D). After several weeks on ruxolitinib she was hospitalized with a viral respiratory infection and ruxolitinib was stopped. Forty-eight hours after ruxolitinib cessation her CD14^+^ monocyte STAT1 protein levels were twice the average of two healthy volunteer CD14^+^ STAT1 levels (data not shown). At the same time, patient CD14^+^ monocyte peak pSTAT1 levels after IFNγ stimulation was 1.8 times the average peak level of the same two healthy volunteer peak pSTAT1 levels (data not shown). Five days after ruxolitinib cessation patient CD14^+^ monocyte STAT1 protein levels were 3.55 times healthy volunteer levels and her pSTAT1 peak level was 3.4 times the healthy volunteer peak level (Figures 5E,F). Patient CD3^+^ cells showed a similar pattern. When ruxolitinib naïve her CD3^+^ cell STAT1 protein levels were increased, while on ruxolitinib her CD3^+^ cell STAT1 protein levels came down to normal. After the treatment was stopped her CD3^+^ STAT1 levels rose again (Figure S4B).

**Figure 6:**
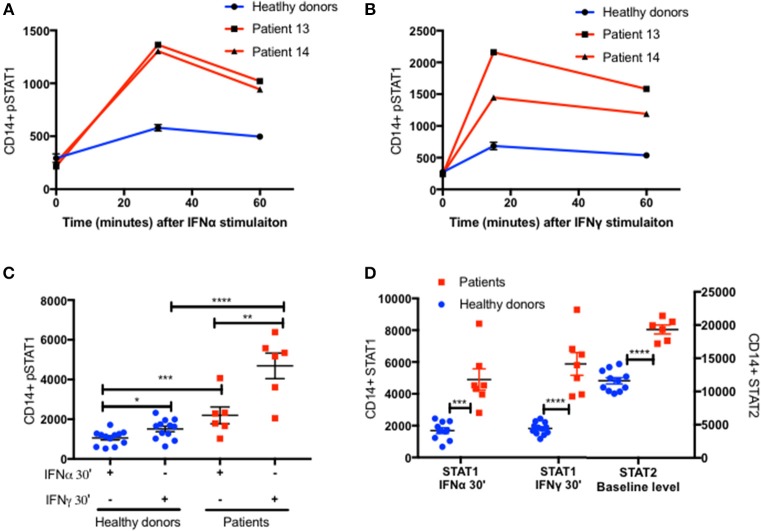
Increased STAT2 protein level in patients CD14^+^ monocytes. Patients 13 and 14 (Red lines) CD14^+^ monocytes pSTAT1 level over 60 min following IFNα **(A)** and IFNγ **(B)** stimulation compared to two healthy donors' average level (blue line), as measured by flow cytometry with an anti pSTAT1 PerCP-Cy 5.5 antibody. Levels are expressed in geometric mean of fluorescence. **(C)** Patients (*n* = 6, red squares) and healthy donors (*n* = 11–12, blue dots) pSTAT1 level 30 min after IFNα or IFNγ stimulation as measured by flow cytometry. **(D)** Patients (*n* = 6, red squares) and healthy donors (*n* = 10–12, blue dots) STAT1 protein level 30 min after IFNα or IFNγ stimulation and baseline STAT2 protein level, as measured by flow cytometry. *P < 0.05; **P < 0.01; ***P < 0.001; ****P < 0.0001, by t test **(C,D)**. Quantitative data represent mean ± SEM **(A–D)**.

Increased pSTAT1 formation is not unique to IFNγ stimulation. Increased levels of pSTAT1 occur following stimulation with IFNγ, IFNα, IL-6, IL-21, and IL-27 (2–5). Previous data showed that pSTAT1 level peaks 30 min after IFNα stimulation and declines gradually thereafter (18). We verified this in two patients and two healthy donors (Figure 6A). Furthermore, we stimulated fresh PBMC of six patients and 12 healthy donors with IFNα and measured both CD14^+^ monocyte STAT1 protein and pSTAT1 at baseline and after 30 min using flow cytometry (Figures 6C,D). In parallel, we used the same patient and healthy donor samples to measure CD14^+^ monocyte STAT1 and pSTAT1 levels following 30 min of IFNγ stimulation (Figures 6B–D). Patient pSTAT1 levels were significantly higher compared to healthy donors following both IFNα and IFNγ stimulation (Figure 6C). However, in both patients and healthy donors, pSTAT1 levels following IFNγ stimulation were significantly higher than following IFNα stimulation (Figure 6C). Thirty minutes after IFNα stimulation, the average patient pSTAT1 level was 2.1 times the healthy donor average level. In contrast, the average patient pSTAT1 level following IFNγ stimulation was 3.1 times the healthy donor average pSTAT1 level (Figure 6C). Mean STAT1 protein levels 30 min after IFNγ stimulation were higher compared to mean STAT1 levels after IFNα stimulation in both patients and healthy donors, however these differences were not significant (*p* = 0.41 and *p* = 0.52, respectively) (Figure 6D). Thirty minutes after IFNα or IFNγ stimulation, the average patient STAT1 levels were 2.8 and 3.2 times the healthy donor average STAT1 level, respectively (Figure 6D).

IFNγ stimulation induces STAT1 phosphorylation and homodimerization (14, 15), whereas IFNα stimulation induces a more complex cascade of STAT1 and STAT2 phosphorylation, heterodimerization, and formation of the IFN-stimulated gene factor (ISGF)3 complex with a third protein, interferon regulatory factor (IRF)9 (15, 19, 20). We measured STAT2 levels in CD14^+^ monocytes of the same six patients and 12 healthy donors at baseline, prior to IFNα stimulation. STAT2 protein levels were significantly higher in patients compared to healthy controls (Figure 6D and Figures S5A,B). Interestingly, the ratio between the patient and healthy donors average STAT2 levels was 1.7, very close to the ratio of 2 between patient and healthy donors average pSTAT1 levels 30 min after IFNα stimulation.

**Figure 7:**
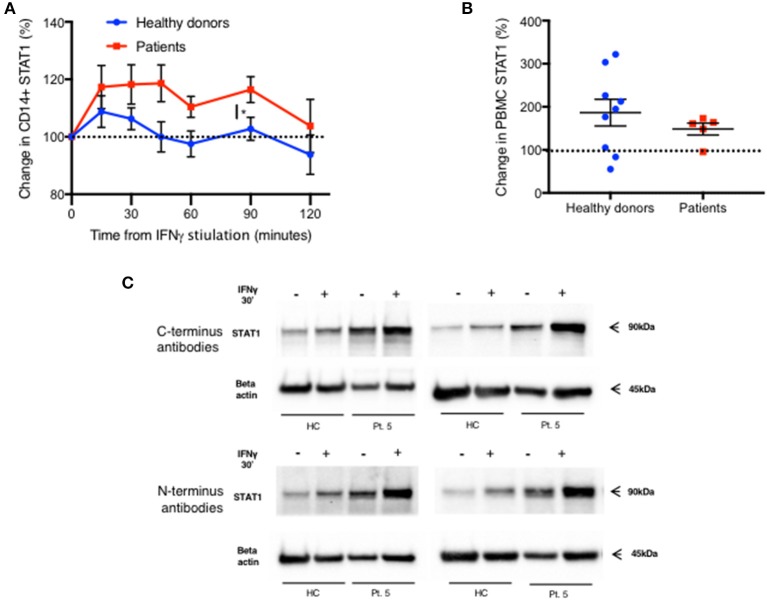
Rapid increase in STAT1 protein level after IFNγ stimulation in both patients and healthy donors. **(A)** Change in STAT1 protein level in CD14^+^ monocytes after IFNγ stimulation in healthy controls (blue line, *n* = 4–16 per time point) and GOF patients (red line, *n* = 3–10 per time point). Change is expressed in percentages from the baseline level. STAT1 level was measured using flow cytometry **(B)** Change in STAT1 protein level in PBMC 30 min after IFNγ stimulation in healthy controls (blue dots, *n* = 9) and GOF patients (red squares, *n* = 5). Change is expressed in percentages from the baseline level. STAT1 level was measured using immunoblotting. **(C)** Patient five STAT1 protein level with and without IFNγ stimulation (30′), compared to a healthy control, as measured by immunoblotting using four different antibodies targeting four different sites of STAT1 protein [two at the C-terminus and two at the N- terminus (see methods)]. The same patient sample was used on three separate membranes. *P < 0.05, by *t*-test. Quantitative data represent mean ± SEM **(A-B)**.

We also noticed an increase in total STAT1 protein levels 15 min after IFNγ stimulation in CD14^+^ monocytes, in both patients and healthy donors (Figure 7A). Fifteen minutes after stimulation average STAT1 protein levels were 109% ± 20 and 117% ± 23 of baseline levels in healthy controls and patients, respectively. After 45 min, healthy volunteer STAT1 protein levels returned to baseline. However, in the patient group the increase in STAT1 protein levels lasted 90 min after stimulation, with total STAT1 protein levels 116% ± 5 of baseline, whereas in healthy controls the level was 103% ± 4 of baseline (*p* < 0.05). By 120 min patient STAT1 protein levels came back to baseline. By immunoblotting, fresh PBMC STAT1 protein levels after 30 min of IFNγ stimulation were 187% ± 31 of baseline levels in healthy volunteers and 148% ± 14 in patients (*p* = 0.4) (Figure 7B), independent of the order of antibody application or epitope (Figure 7C).

**Figure 8:**
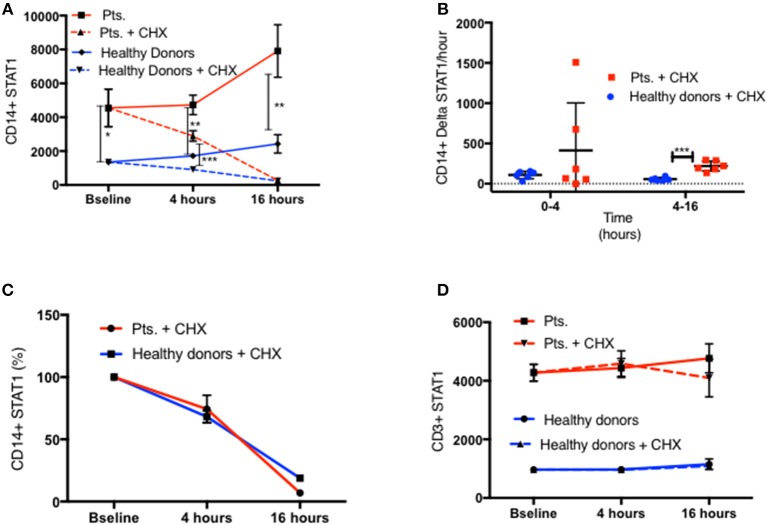
Similar STAT1 degradation rate in patients and healthy donors. **(A)** CD11b^+^/CD14^+^/CD64^+^ live monocytes STAT1 protein level in fresh PBMC of patients (red lines, *n* = 6) and healthy controls (blue lines, *n* = 8) at baseline and after 4 and 16 h with cycloheximide (CHX) 100 ng/ml (dotted lines) and without cycloheximide (solid lines), as measured by flow cytometry. Levels are expressed in geometric mean of fluorescence. **(B)** Average absolute decrease in STAT1 protein per hour in the monocytes of healthy donors (blue dots, *n* = 8) and patients (red squares, *n* = 6), during the first 4 h (0–4) and the last 12 h (4–16) of cycloheximide incubation. **(C)** Rate of decrease (%) in STAT1 protein level in healthy donor (blue line, *n* = 8) and patient (red line, *n* = 6) monocytes during the first 4 h (0–4) and the last 12 h (4–16) of cycloheximide incubation. **(D)** CD3^+^ lymphocyte STAT1 protein level in fresh PBMC of patients (red lines, *n* = 6) and healthy controls (blue lines, *n* = 8) at baseline and after 4 and 16 h with cycloheximide (CHX) 100 ng/ml (dotted lines) and without cycloheximide (solid lines) as measured by flow cytometry. Levels are expressed in geometric mean of fluorescence. *P < 0.05; **P < 0.01; ***P < 0.001, by *t*-test. Quantitative data represent mean ± SEM **(D)**.

One possible explanation for the increased STAT1 protein levels in GOF patients is resistance of the mutant STAT1 protein to degradation. Since STAT1 drives its own transcription (21), we queried whether resistance of the protein to degradation could lead to higher levels of STAT1 protein that in turn could lead to higher *STAT1* transcription. Therefore, we examined STAT1 protein degradation and *STAT1* mRNA expression in GOF patient and healthy volunteer cells. We measured CD14^+^ monocytes and CD3^+^ STAT1 levels at baseline and after 4 and 16 h of incubation with and without cycloheximide 100ng/ml in six patients (four different STAT1 GOF pathogenic variants) and eight healthy volunteers. As seen in Figure 8A in both patient and control CD14^+^ monocytes, STAT1 protein levels increased during incubation in culture media. At all time points patient mean CD14^+^ monocyte STAT1 levels were significantly higher than healthy volunteer mean levels (2.75–3.3 times healthy volunteer mean level) (Figure 8A). Adding cycloheximide to the culture resulted in a decrease in CD14^+^ monocyte STAT1 protein levels in both groups (Figure 8A). During the first 4 h the mean absolute decrease in STAT1 protein level in the patient group was not significantly different from the mean absolute decrease in the healthy volunteer group (Figure 8B). Over hours 4–16 of cycloheximide incubation, the mean absolute decrease in STAT1 protein was 3.9 times faster in the patient group than in the healthy volunteer group (*p* = 0.0001) (Figure 8B). When we looked at the average rate of STAT1 degradation expressing STAT1 level as a percentage of baseline there was no difference between patient and healthy control cells (Figure 8C). Patient mean CD3^+^ STAT1 levels were 4.1–4.6 times the healthy volunteer CD3^+^ STAT1 levels at all time points measured (baseline, 4 and 16 h; *p* < 0.0001 for all) (Figure 8D). In contrast to the change in STAT1 over tine in CD14^+^ monocytes, CD3^+^ cell STAT1 levels did not change over time regardless of the addition of cycloheximide in both patients and healthy volunteers (Figure 8D).

**Figure 9:**
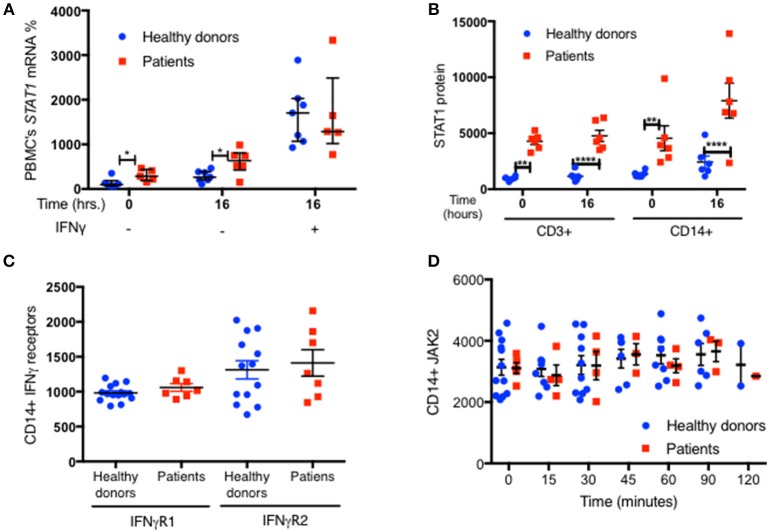
Increased *STAT1* mRNA expression in GOF PBMC. **(A)** Average fresh PBMC *STAT1* mRNA relative expression at baseline and after 16 h *in vitro* incubation with and without IFNγ stimulation, in healthy controls (blue dots, *n* = 7) and GOF patients (red squares, *n* = 6). Levels are expressed in percentages of same day healthy controls median *STAT1* mRNA relative expression. Each dot or square represent an average of 1–3 biological replicates. *Beta actin* was used as the normalizing gene. **(B)** CD3^+^ and CD14^+^ cells STAT1 protein level in the samples used for the RNA extraction of the seven healthy controls and six GOF patients in **(A)**. STAT1 levels were measured by flow cytometry and are expressed in geometric mean of fluorescence. **(C)** Levels of IFNγ receptors (R) 1 and 2 in healthy controls (blue dots; *n* = 13–15) and GOF patients (red squares, *n* = 7), as measured by flow cytometry. Data is represented in geometric mean of fluorescence. Each dot or square represents the average of two technical duplicates. **(D)** JAK2 protein level in healthy controls (blue dots, *n* = 2–12 per time point) and GOF patients (red square, *n* = 1–5 per time point) at rest (time o) and up to 2 h of IFNγ stimulation, as measured by flow cytometry. Data is represented in geometric mean of fluorescence. *P < 0.05; **P < 0.01; ****P < 0.0001, by *t*-test, Quantitative data represent mean ± SEM.

We measured *STAT1* mRNA expression in fresh PBMC from 6 GOF patients with four different mutations and seven healthy controls. Lehtonen et al., demonstrated that IFNγ stimulation of PBMC induced an increase in *STAT1* mRNA, which peaked around 8–16 h and plateaued for an additional 34 h (21). We looked at PBMC *STAT1* mRNA levels immediately after separation of PBMC from fresh whole blood, and again after 16 h of incubation in culture media with and without IFNγ stimulation. Baseline fresh PBMC median *STAT1* mRNA expression was 2.9 times higher in six tested patients than seven healthy volunteers (*p* = 0.03) (Figure 9A). After 16 h in culture media the same six patient PBMC had higher levels of *STAT1* mRNA than the same seven healthy volunteers, a median 633 vs. 265%, compared to the healthy controls respective baseline (*p* = 0.035) (Figure 9A). STAT1 protein levels were significantly higher at baseline (time 0) and after 16 h in culture media, in both CD14^+^ monocytes and CD3^+^ cells from the six patients compared to the seven healthy volunteers (Figure 9B). In both healthy volunteers and GOF patients there was a substantial increase in *STAT1* mRNA after IFNγ stimulation (Figure 9A). Interestingly, after 16 h of IFNγ stimulation there were no differences in relative *STAT1* mRNA expression between patients and healthy volunteers (Figure 9A).

To exclude the possibility that other components of the JAK-STAT pathway were contributing to the high pSTAT1 levels in *STAT1* GOF mutations, we looked at IFNγ receptors 1 and 2 and JAK2 levels. We found no significant differences in any of these protein levels between patient and control CD14^+^ monocytes (Figures 9C,D and Figures S5A,B).

## Discussion

It is widely agreed that cells containing STAT1 GOF pathogenic variants have much higher and more enduring levels of pSTAT1, which is tied directly to the mutations (2–5, 10–12). However, the mechanistic causes and effects of the elevated pSTAT1 levels in *STAT1* GOF have been complex to decipher. While delayed dephosphorylation has been hypothesized by us and others (2–5, 11, 12), the mediators of this have been difficult to define. While it is true that levels of pSTAT1 are higher with cytokine stimulation, our data show that this is not likely to be due to defective dephosphorylation. In fact, in absolute numbers, dephosphorylation was faster in *STAT1* GOF patients than that in controls. We first observed and reported normal STAT1 dephosphorylation in two patients with STAT1 GOF pathogenic variants, c.1057G>A E353K and c.796G>A V266I, in 2015 (22). Since then we confirmed our findings and verified them in an additional 13 patients and nine mutations in multiple independent experiments comparing patients with 40 healthy donors. Sobh et al. (13), Weinacht et al. (23), and Meesilpavikkai et al. (24), have also newly reported *STAT1* GOF pathogenic variants in the SH2 (p.H629Y and p.V653I) and linker (p.E545K) domains with high pSTAT1 levels and normal dephosphorylation. Our current data show that this normal dephosphorylation rate also applies to mutations in the coiled-coil and DNA binding domains (1–4, 11, 12).

Our demonstration of increased STAT1 protein levels in all 10 GOF mutations tested provides a new explanation for the high and prolonged levels of pSTAT1 found in GOF mutations. The strong correlation between total STAT1 protein levels and peak pSTAT1 levels following IFNγ stimulation, both in patients and controls, and the fact that the patient pSTAT1/STAT1 ratio is the same as in healthy controls, supports the hypothesis that high resting and stimulated total STAT1 protein levels are the background against which these high pSTAT1 levels occur with GOF mutations.

The finding that treatment with the oral JAK inhibitor ruxolitinib normalized both STAT1 protein and pSTAT1 levels in a patient with GOF mutation, and that both total STAT1 protein and pSTAT1 levels increased in proportion after ruxolitinib treatment was stopped, further supports our hypothesis that STAT1 protein level is the critical factor driving peak pSTAT1 levels in CD14^+^ monocytes. Therefore, it appears that the reason for increased pSTAT1 levels in GOF patients is increased total STAT1 protein levels. Additional support for this hypothesis comes from Tabellini et al., who reported high levels of STAT1 protein in NK cells from seven patients carrying five different coiled-coil or DNA binding domain STAT1 GOF pathogenic variants, as measured by flow cytometry (25) and Bernasconi et al. who reported high levels of STAT1 protein in CD14^+^ monocytes, which correlated with high pSTAT1 levels, in 8 patients STAT1 pathogenic variants (26).

The fact that IFNγ receptors 1 and 2 and JAK2 levels in CD14^+^ monocytes were normal in the tested GOF patients, argues against a role of these specific other components of the STAT1 signal cascade causally contributing to the high STAT1 levels in GOF patients and suggests that in IFNγ stimulated CD14^+^ monocytes the critical factor determining pSTAT1 level is total STAT1 protein level itself. We did not specifically look at JAK1 levels. IFNα stimulation also led to increased pSTAT1 formation in GOF mutations, but to a lesser extent than IFNγ. In addition, STAT2 protein levels were elevated at baseline in patients with GOF mutations. The ratios between patient to healthy donor IFNα induced pSTAT1 level to patient and healthy donor STAT2 baseline level were similar. We did not explore the mechanisms behind the increased STAT2 level in GOF patients, but this is an important future research direction, especially given the difficulties with viral infections in STAT1 GOF disease (11).

There were several possible explanations for the high total STAT1 protein levels in GOF mutations. The mutations may have made the protein relatively resistant to degradation, creating a cycle in which more STAT1 molecules are available for phosphorylation and signaling, thereby driving type I and II interferon target transcription. Alternatively, the mutations might drive increased STAT1-associated transcription in general (e.g., by prolonged STAT1 DNA binding). Since STAT1 participates in the regulation of its own transcription (21), there might be increased transcription and translation by the mutated STAT1 protein resulting in higher total STAT1 protein levels. Our findings do not support the hypothesis that STAT1 GOF proteins have delayed degradation, at least not in the tested mutations. Absolute STAT1 degradation was either normal or faster than normal in CD14^+^ cells in the tested GOF patients. Cycloheximide had no effect on CD3^+^ T cells STAT1 (Figure 8D) or CD3 receptor levels (data not shown), whereas CD14^+^ positive cells showed a fast and significant decrease in both STAT1 (Figures 8A–C) and CD14 receptor (Data not shown) levels under cycloheximide treatment. A previous report of cycloheximide and STAT1 levels in lymphoblastoid cell lines (27) found significant STAT1 degradation within 3 h of cycloheximide treatment, while in cells carrying a Fanconi anemia gene, STAT1 protein levels stayed stable for 17 h after cycloheximide treatment. The lack of decrease in CD3^+^ receptor levels suggests that primary peripheral CD3^+^ cells are not terribly sensitive to cycloheximide for up to 16 h, while CD14^+^monocytes are more rapidly susceptible.

Some of our findings support the hypothesis that the increased STAT1 protein levels are a result of increased *STAT1* transcription. *STAT1* relative mRNA expression was significantly increased in fresh patient PBMC and after 16 h in culture. However, *STAT1* mRNA was not higher after 16 h of IFNγ stimulation. Tabellini et al. had similar findings of increased *STAT1* mRNA in resting NK cells of one patient with STAT1 gain of function mutation (c.801T>A, p.A267V) (25). It is possible that the increased resting STAT1 level in the GOF mutations is due to transcription that is not IFNγ dependent. Overall, the exact mechanism(s) behind the increased *STAT1* transcription in STAT1 GOF mutations are yet to be determined.

These observations make it clear that there are likely different ways in which total STAT1 levels can be brought about. Therefore, although we surmise that elevated total STAT1 is common to the GOF mutations, it is formally possible that different GOF STAT1 mutations may have different mechanisms, such as impaired degradation, increased transcription, or regulation of other pathways.

We are aware that these findings contradict the previous narrative regarding STAT1 GOF mutations (2–5), as proffered by ourselves and others. We believe that the reasons for these previous interpretations lay in the methods used, including staurosporine as the sole kinase inhibitor. The initial publications on STAT1 GOF mutations relied mainly on immunoblotting assays (2–4). Taylor et al. cited the weakness of immunoblotting as a quantitative tool (6, 7) and emphasized the narrow linear range of this method. In contrast, flow cytometry allowed us to measure events at a single cell level, to differentiate between live and dead cells, and to focus on specific cell populations (28, 29) over a 5-log dynamic range (29). It also enabled the study of pSTAT1 kinetics in ways that were less cumbersome than immunoblot.

The observed increase in STAT1 protein levels 30 min following IFNγ stimulation was unexpected, and poses new questions regarding STAT1 transcription, translation and regulation. Previous data showed that in human PBMC, *STAT1* mRNA expression starts to increase at 4 h and reaches a plateau 8–16 h after stimulation with IFNγ (21), which lasts up to 42 h. Therefore, the cause of the rapid increase in STAT1 protein following stimulation is yet to be defined.

The increase in STAT1 following IFNγ stimulation as determined by immunoblotting using fresh PBMC was higher than that observed with flow cytometry on CD14^+^ cells. The reasons for these differences are likely multi-factorial, including that the immunoblot was performed on PBMC, whereas the flow cytometry was performed on CD14^+^ monocytes. Other cell populations in PBMC such as B, T or NK cells, may have more significant increases in STAT1 protein levels following IFN-gamma stimulation.

Our current findings are further supported by three new *STAT1* GOF mutations with normal dephosphorylation (13, 23, 24), as well as the finding of increased STAT1 protein levels (25, 26). We believe that this new body of evidence allows better understanding of the mechanism behind STAT1 GOF mutations and may lead to new research directions. The underlying causes of the increased *STAT1* transcription associated with GOF mutations remain unclear. Further investigation and experiments, such as ChIP-seq, will help clarify the mechanisms behind our findings. Better mechanistic understanding of STAT1 signaling and transcription in general and in GOF mutations in particular may help identify specific therapies for specific complications. Further, elevated resting and stimulated STAT1 protein levels might be a simple screening test for STAT1 GOF mutations.

## Ethics Statement

This study was carried out in accordance with the recommendations of NIH IRB committee with written informed consent from all subjects. All subjects gave written informed consent in accordance with the Declaration of Helsinki. The protocol was approved by the NIAID, NIH, IRB committee.

## Author Contributions

OZ, LR, AH, and SH designed research. OZ, PO, LR, and HK performed research. AF, GU, and CZ supplied patient care. OZ, PO, LR, HK, KH, DS, and LD collected data. OZ, PO, LR, GU, CZ, SR, HK, AH, and SH analyzed and interpreted data. OZ and SH performed statistical analysis. OZ, PO, AF, GU, SR, HK, ES, AH, and SH wrote the manuscript.

### Conflict of Interest Statement

The authors declare that the research was conducted in the absence of any commercial or financial relationships that could be construed as a potential conflict of interest.

## Supplementary Material

Supplementary Data Sheet 1 Supplementary method section.

Supplementary Presentation 1 Immunoblotting raw data.
