# Supplementary material for: STAT1 Gain-of-Function Mutations Cause High Total STAT1 Levels With Normal Dephosphorylation
Source: Front Immunol. 2019 Jul 10;10:1433. doi: 10.3389/fimmu.2019.01433 (PMC6635460; doi:10.3389/fimmu.2019.01433)
Supplement: Supplementary Data Sheet 1 — Supplementary method section. [file Data_Sheet_1.docx]

Supplementary Method Section

**Immunoblotting assays**

Immunoblotting was used to determine STAT1 protein and pSTAT1 levels in both patient and healthy donor PBMC. Isolated fresh PBMC were stimulated for 30 minutes with IFNγ 400U/ml. Cell activation was blocked with cold PBS for 5 minutes. Cells were washed twice at 4ᵒC and were lysed in RIPA Lysis and Extraction Buffer (Thermo Fisher Scientific 89900) with protease and phosphatase inhibitors (Thermo Fisher Scientific 78430 and 78420, respectively), on ice for 15 minutes. Lysates were sonicated and then centrifuged at 14,000xg for 15 minutes at 4ᵒC. Protein concentrations were measured by Bradford protein assay (Bio-Rad). Samples were prepared with 4X Laemmli buffer (Bio-Rad) and heated at 100ᵒC for 5 minutes and were subjected to sodium dodecyl sulfate polyacrylamide gel electrophoresis (SDS-PAGE), using 10% polyacrylamide gels, with a 4% stacking layer. Samples were run at 0.5mA, in running buffer (NuPAGE®) using XCell SureLock™ Mini-Cell Electrophoresis System (Thermo Fisher). Electrophoretic transfer of protein to 0.2 µm nitrocellulose membranes (Thermo Fisher), was performed using XCell II™ Blot Module (Thermo Fisher) in transfer buffer (NuPAGE®) at 30V for 1 hour on ice. Transfer of proteins was confirmed by Ponceau staining (Bio-Rad). Membranes were blocked in Tris-Buffered Saline with Tween-20 (TBS-T) and 5% dry milk for 1 hour at room temperature, washed with TBS-T and incubated over night at 4ᵒC with primary antibodies directed against either pSTAT1 (pY701; Cell Signaling, 9167), total STAT1 (Cell Signaling 9172, 14994, 14995 or Becton Dickinson 610115), or beta actin (Cell Signaling 4967). Membranes were washed and incubated for one hour with anti-rabbit/Mouse IgG, HRP-linked Antibody (GE, Cell Signaling or Jackson Immuno Research), at room temperature, washed again and incubated with Clarity™ Western ECL Substrate (Bio-Rad) for 5 minutes.

Data were acquired using ChemiDoc MP imaging system (Bio Rad) and analyzed using Image Lab software (© 2014 Bio-Rad Laboratories; version 5.2.1).

To optimize immunoblotting for quantitation, we determined the linear range for STAT1 and beta actin antibodies that were used (6-7). Both STAT1 and beta actin had low and narrow linear ranges between 2 to 16mcg total protein (Figure S6). Hence, we loaded only 10-15mcg total protein per sample.
