# Supplementary material for: STAT1 Gain-of-Function Mutations Cause High Total STAT1 Levels With Normal Dephosphorylation
Source: Front Immunol. 2019 Jul 10;10:1433. doi: 10.3389/fimmu.2019.01433 (PMC6635460; doi:10.3389/fimmu.2019.01433)
Supplement: Supplementary Presentation 1 — Immunoblotting raw data. [file Presentation_1.PPTX]

## Slide 1
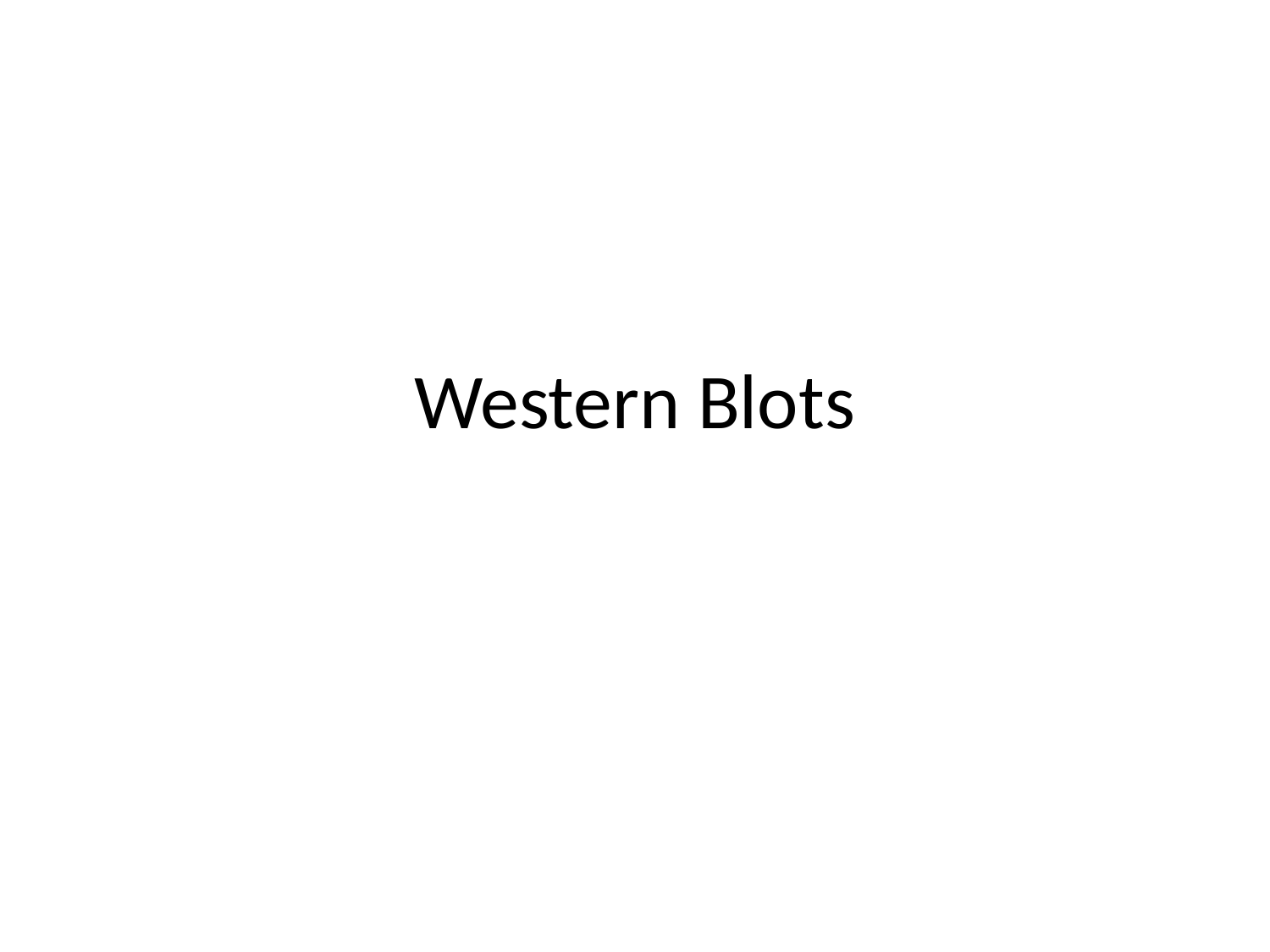

# Western Blots

## Slide 2
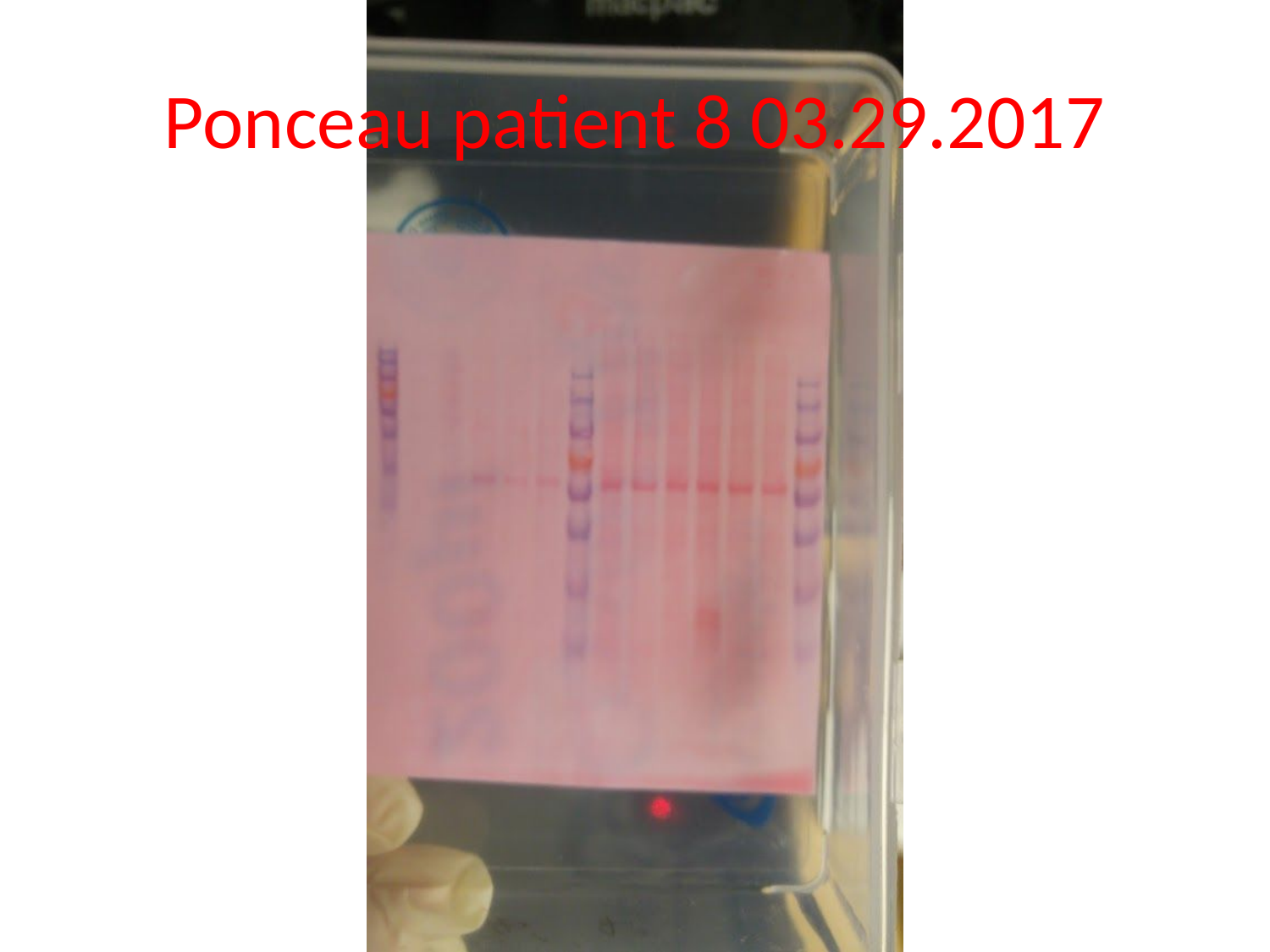

# Ponceau patient 8 03.29.2017

## Slide 3
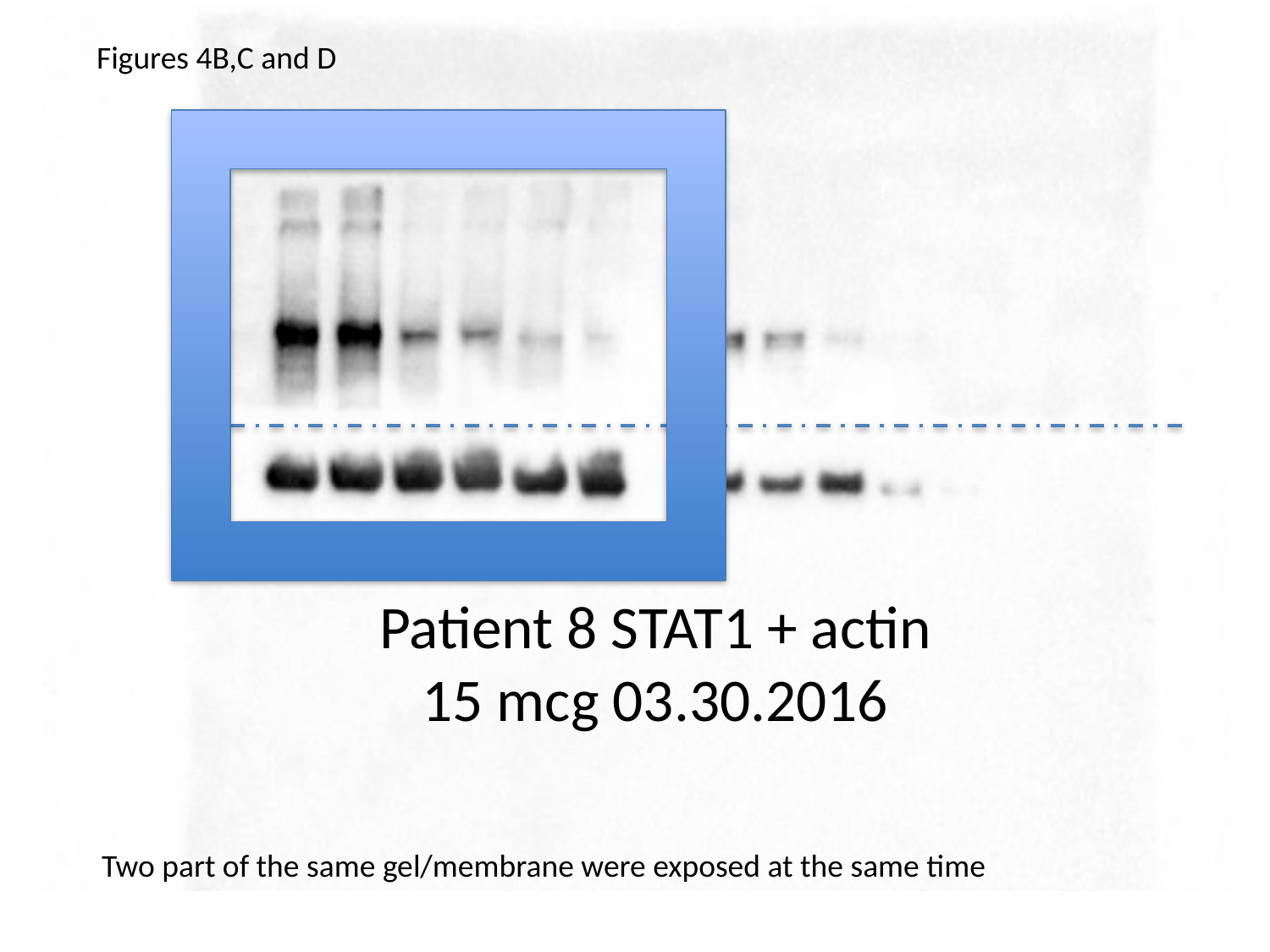

Figures 4B,C and D
# Patient 8 STAT1 + actin 15 mcg 03.30.2016
Two part of the same gel/membrane were exposed at the same time

## Slide 4
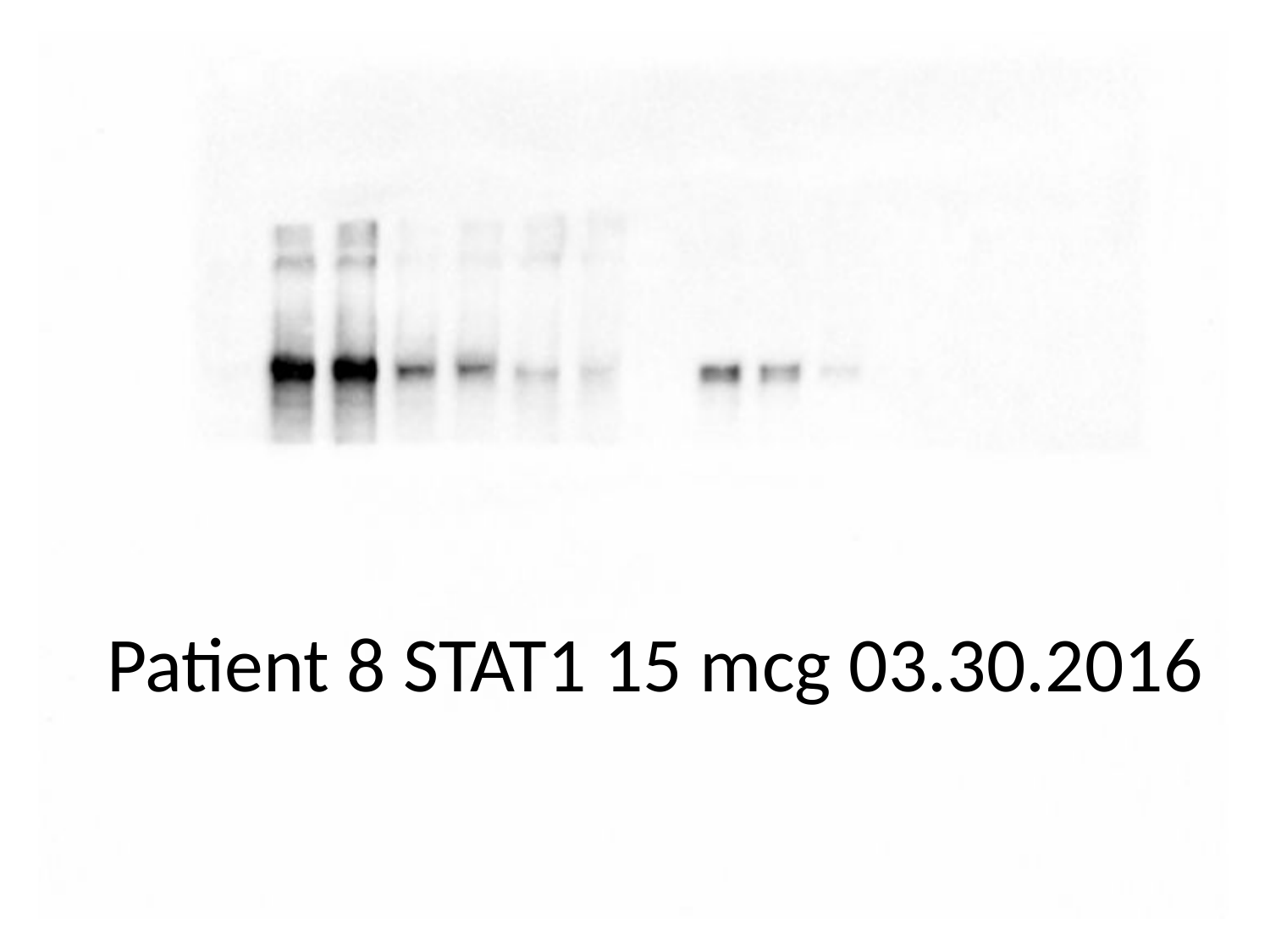

# Patient 8 STAT1 15 mcg 03.30.2016

## Slide 5
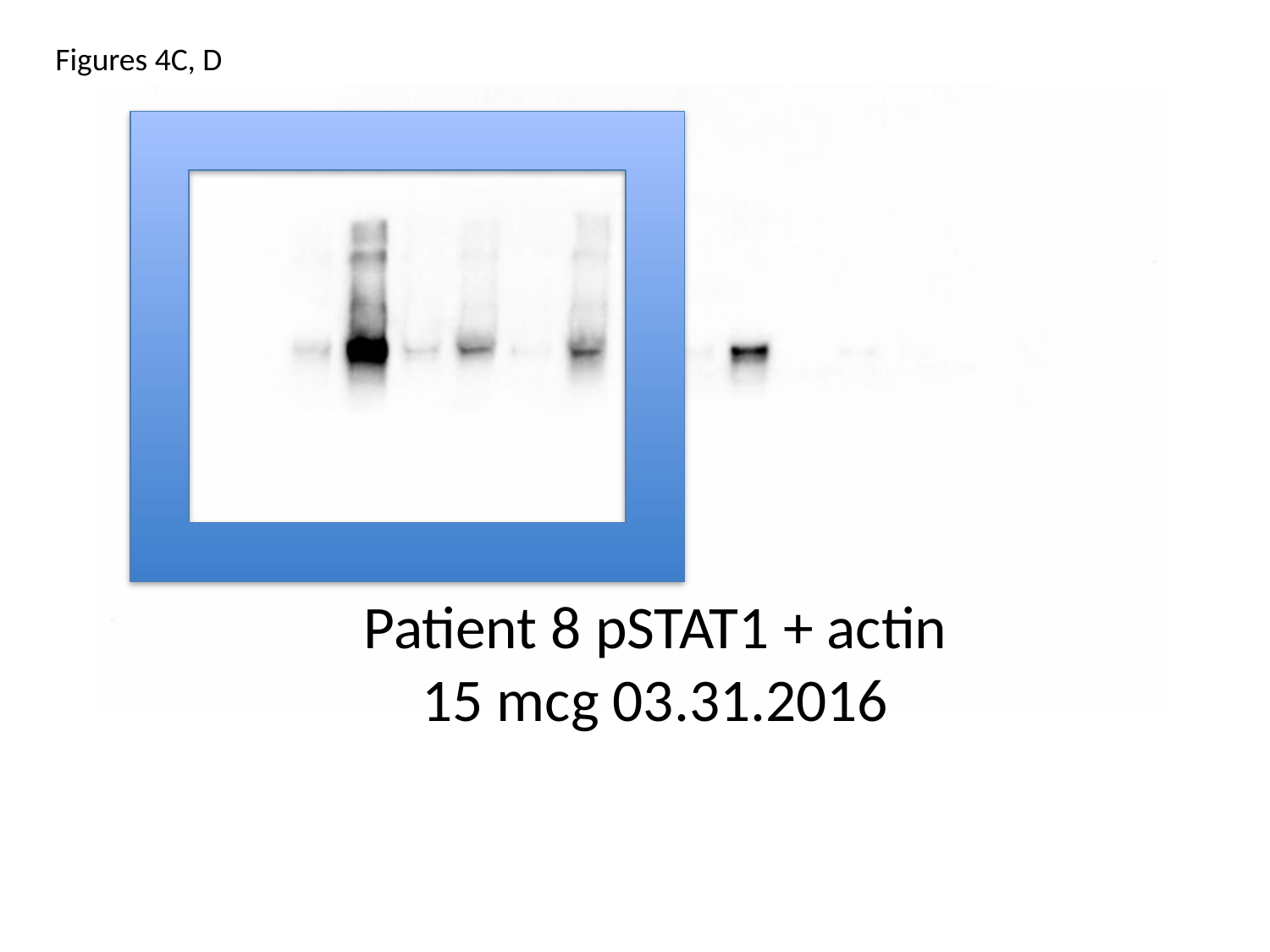

Figures 4C, D
# Patient 8 pSTAT1 + actin 15 mcg 03.31.2016

## Slide 6
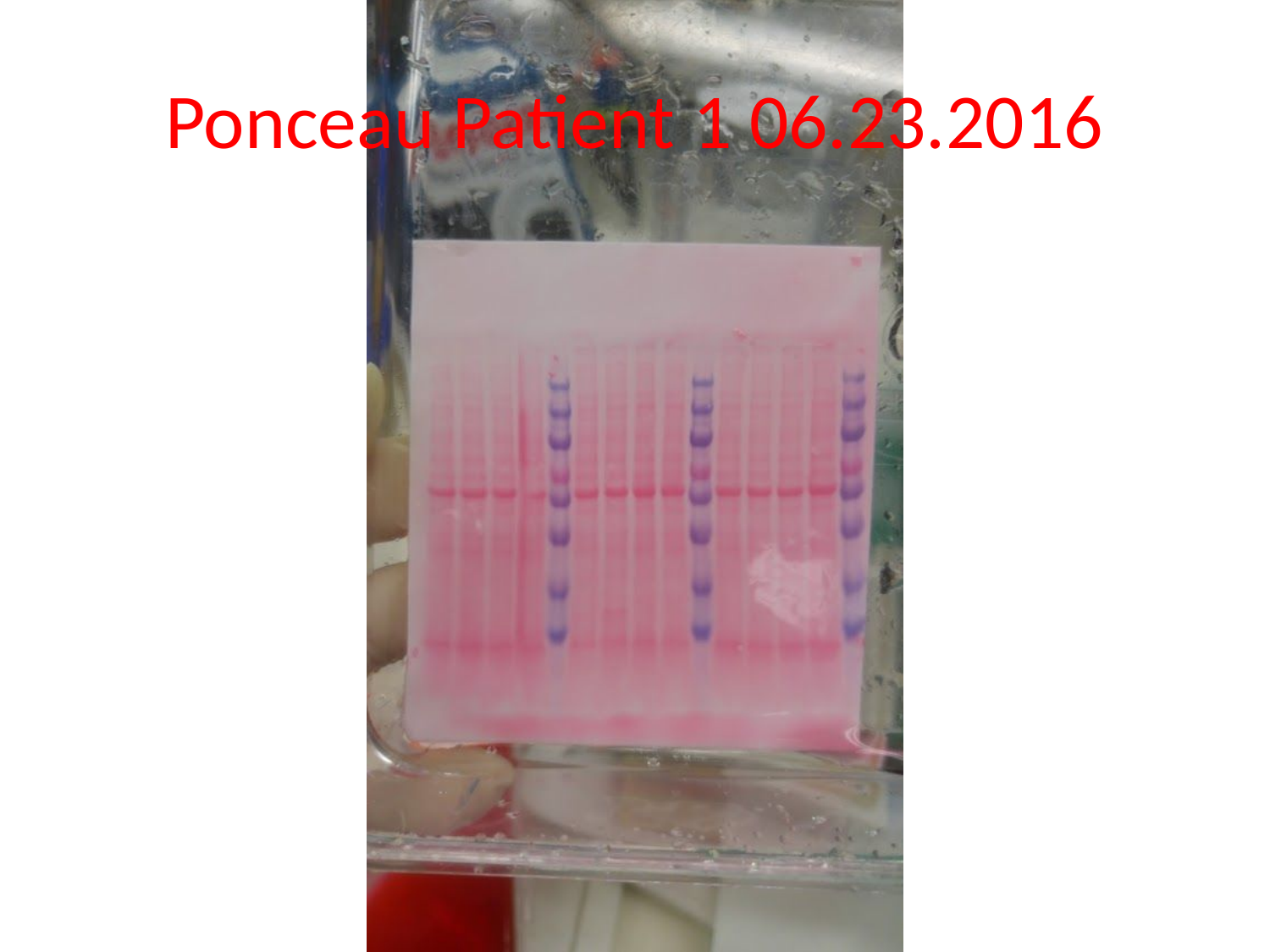

# Ponceau Patient 1 06.23.2016

## Slide 7
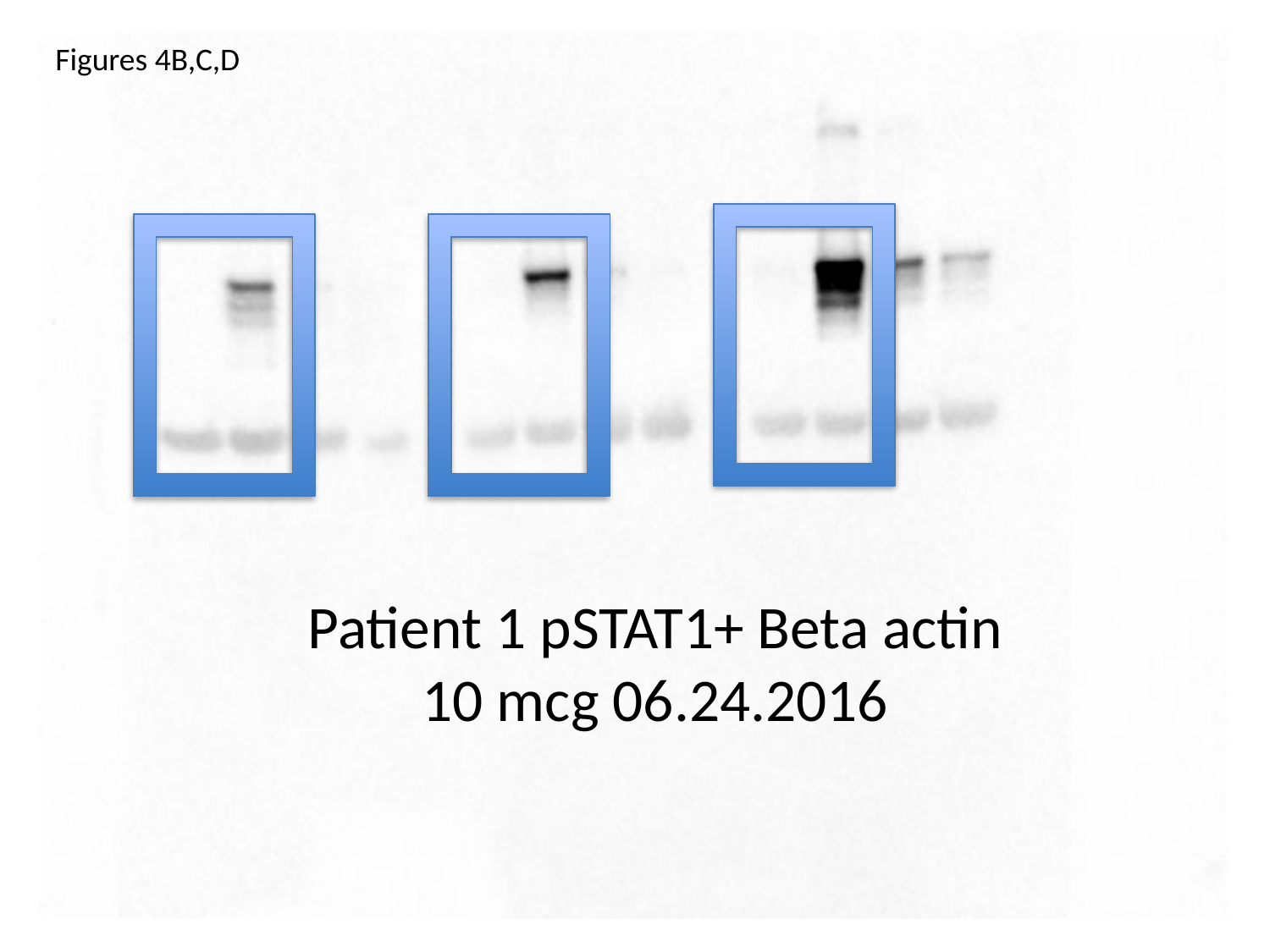

Figures 4B,C,D
# Patient 1 pSTAT1+ Beta actin 10 mcg 06.24.2016

## Slide 8
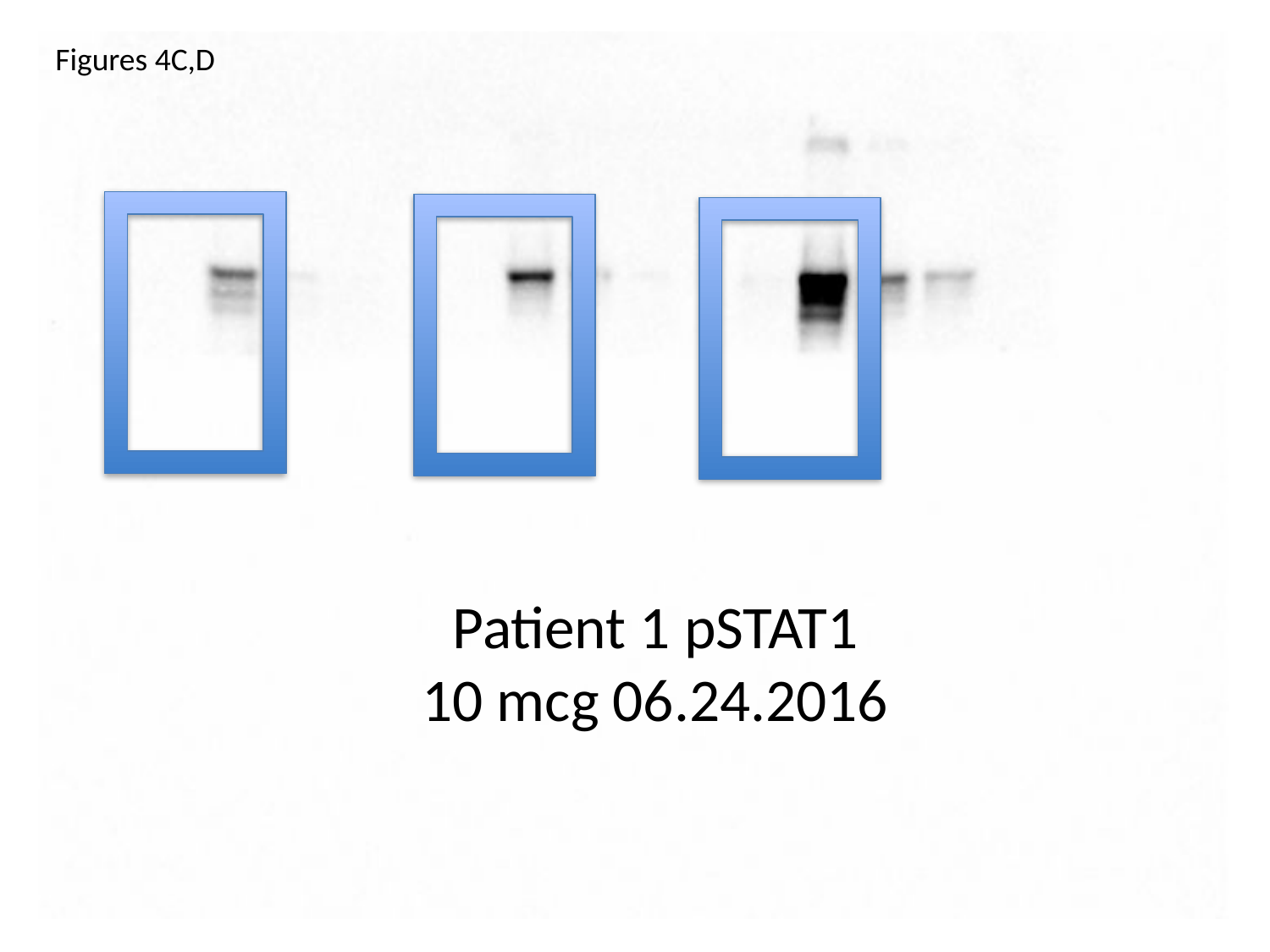

Figures 4C,D
# Patient 1 pSTAT1 10 mcg 06.24.2016

## Slide 9
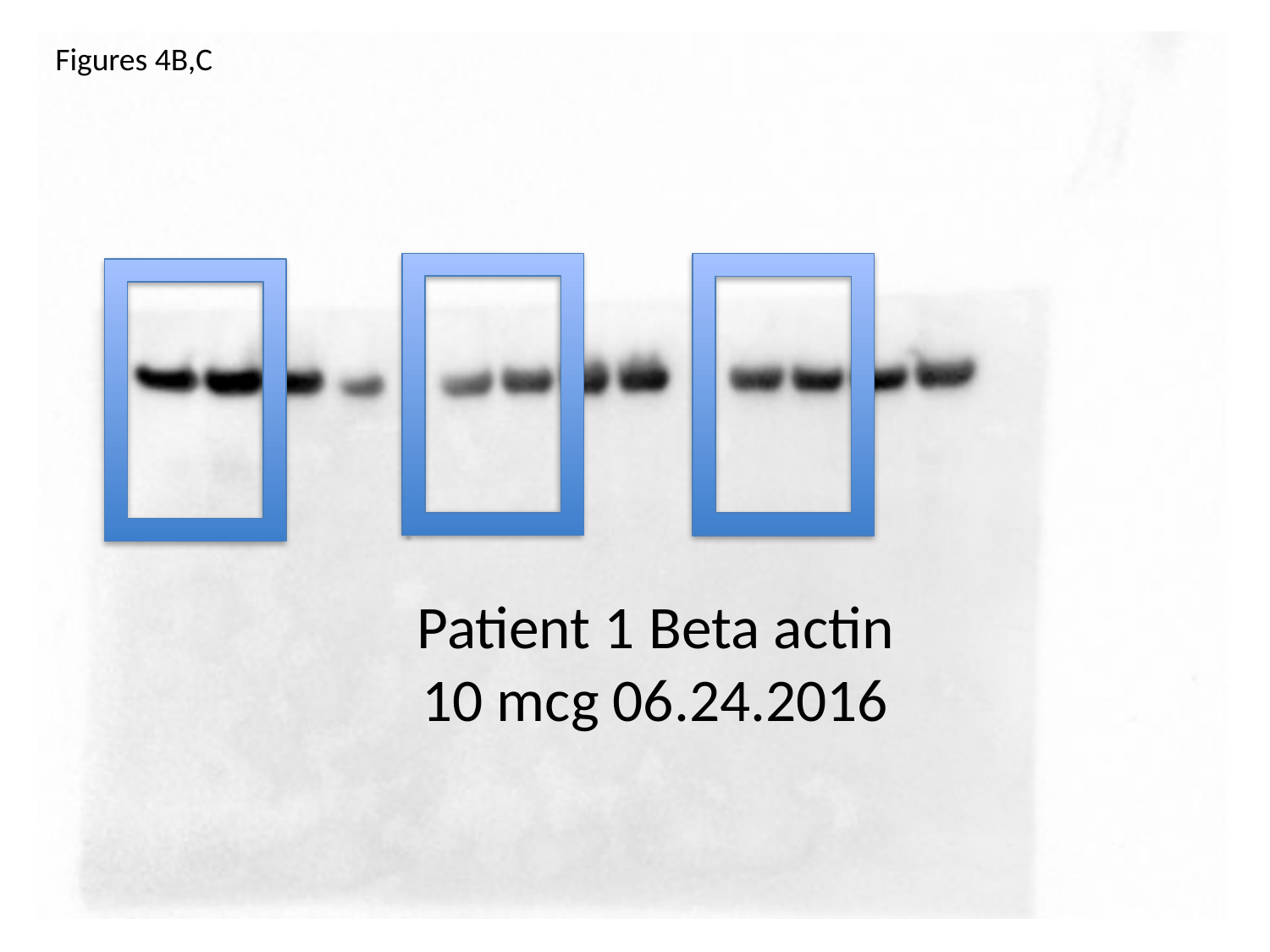

Figures 4B,C
# Patient 1 Beta actin 10 mcg 06.24.2016

## Slide 10
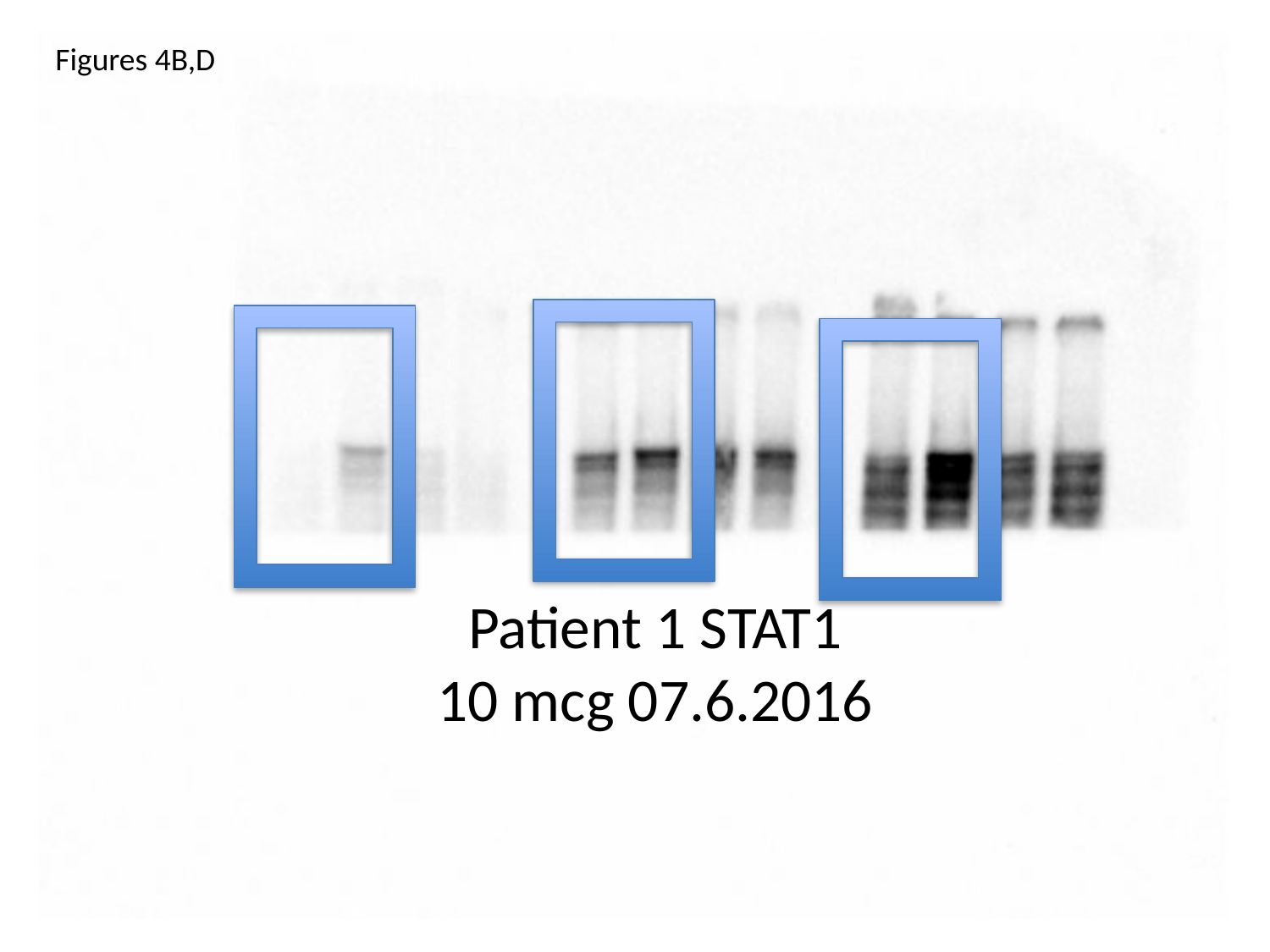

Figures 4B,D
# Patient 1 STAT1 10 mcg 07.6.2016

## Slide 11
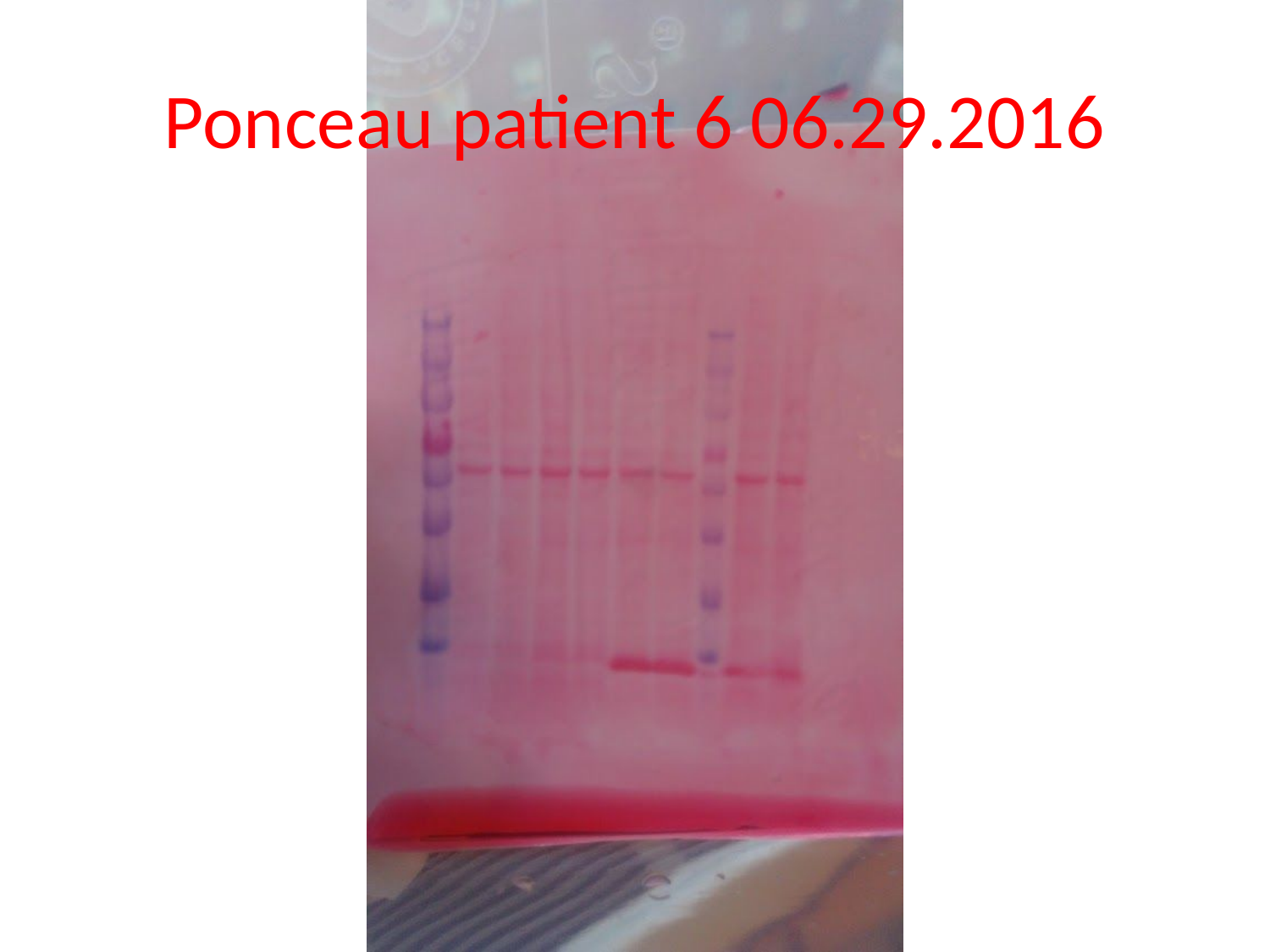

# Ponceau patient 6 06.29.2016

## Slide 12
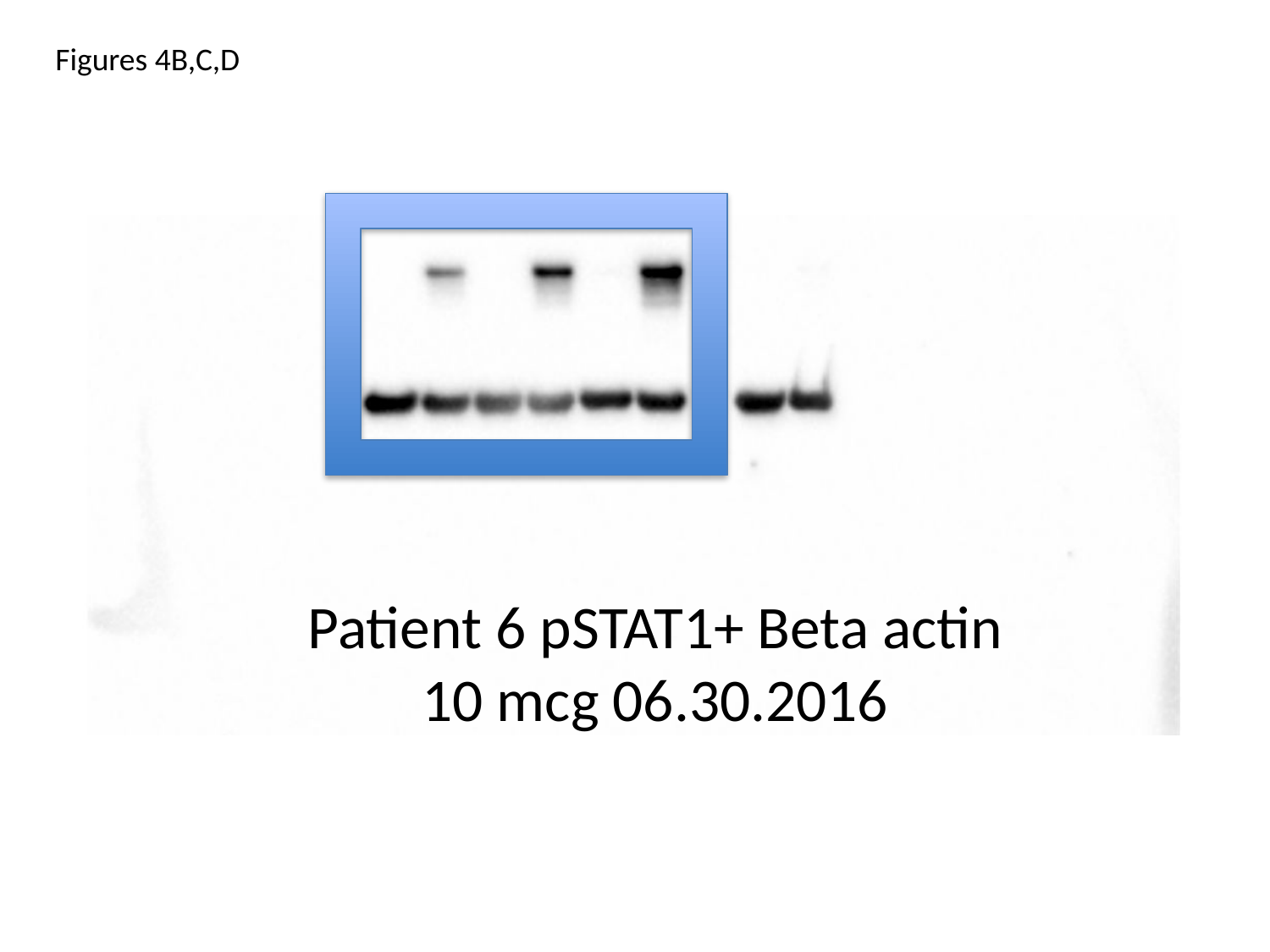

Figures 4B,C,D
# Patient 6 pSTAT1+ Beta actin 10 mcg 06.30.2016

## Slide 13
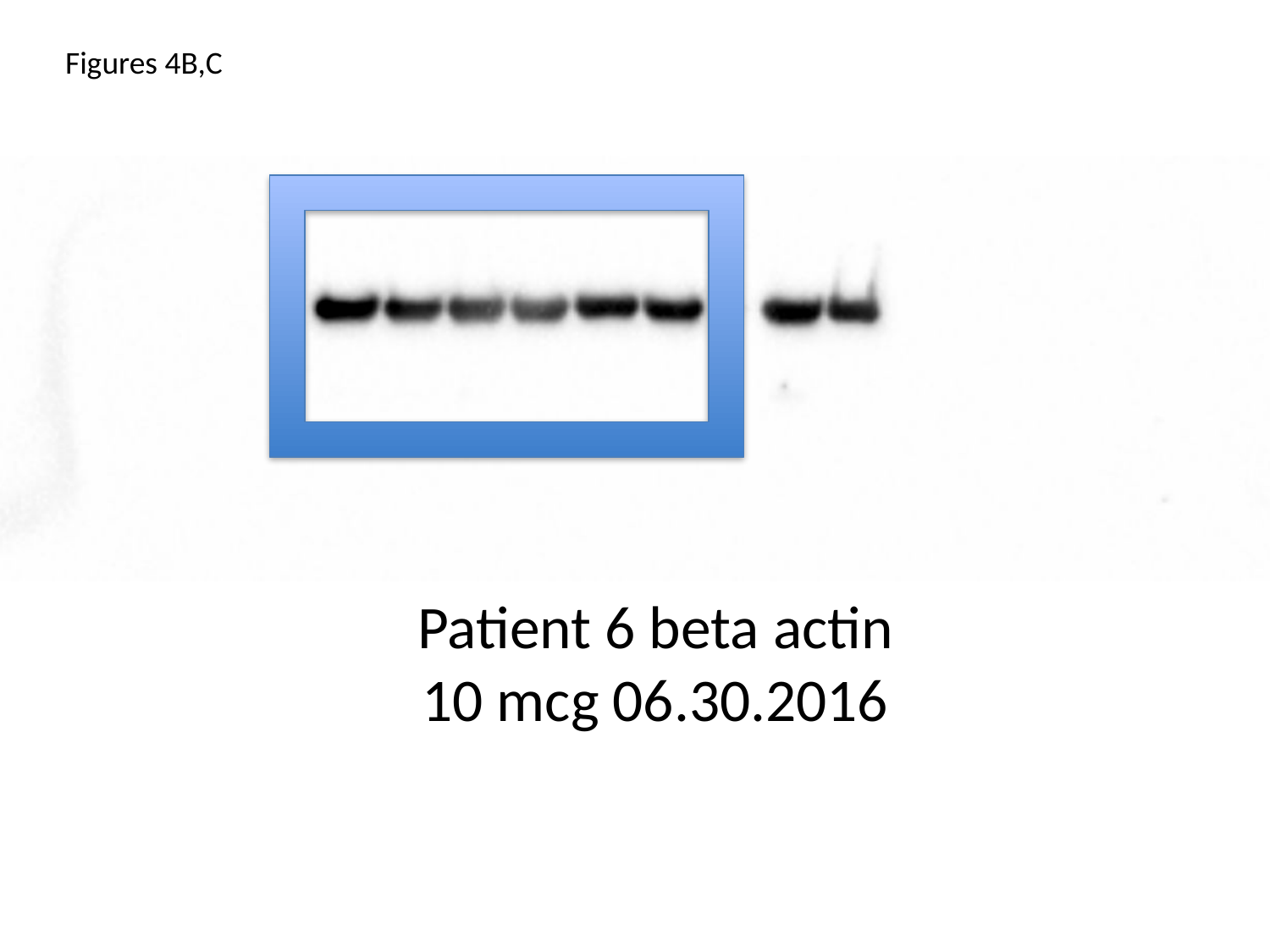

Figures 4B,C
# Patient 6 beta actin 10 mcg 06.30.2016

## Slide 14
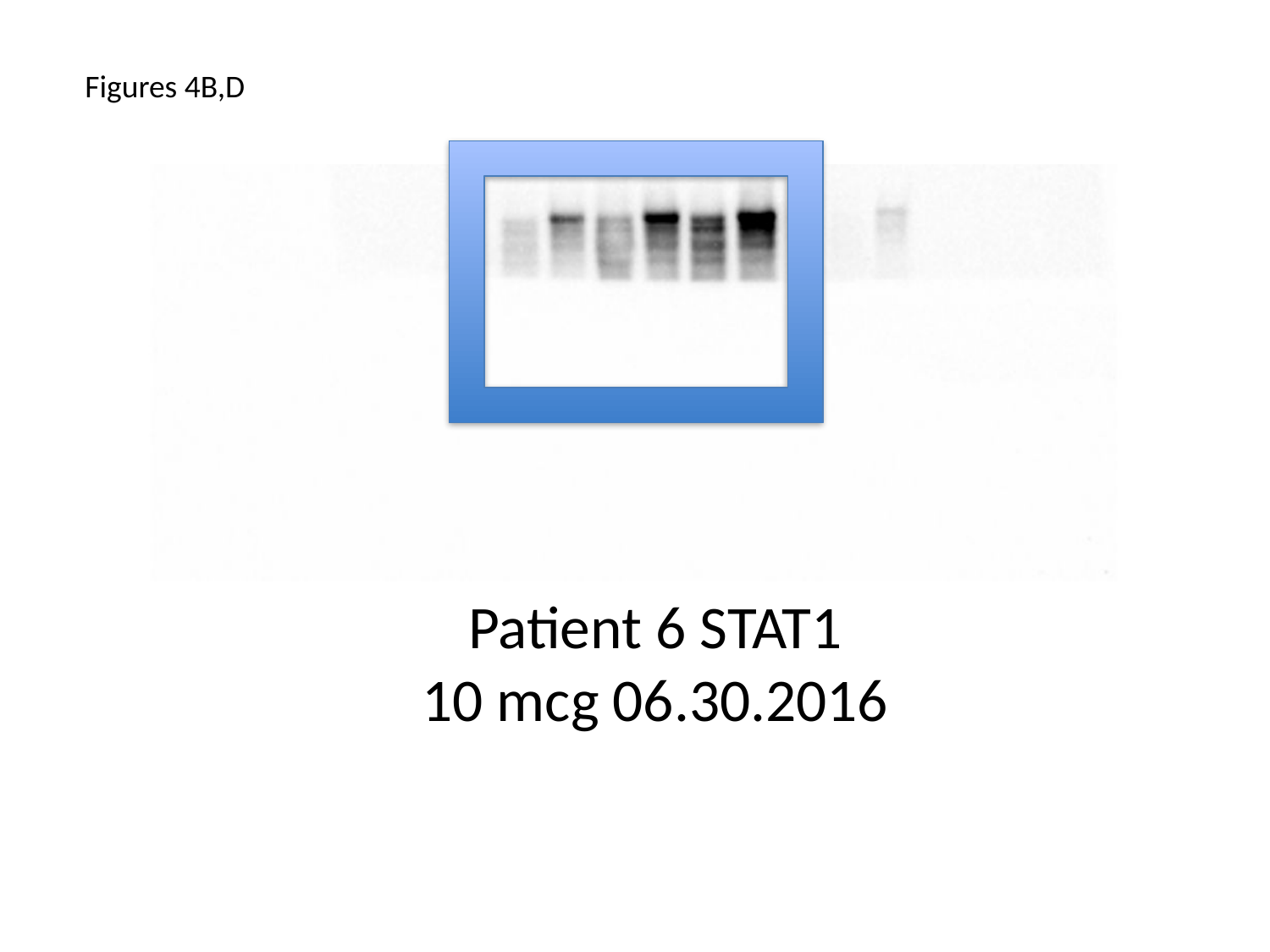

Figures 4B,D
# Patient 6 STAT1 10 mcg 06.30.2016

## Slide 15
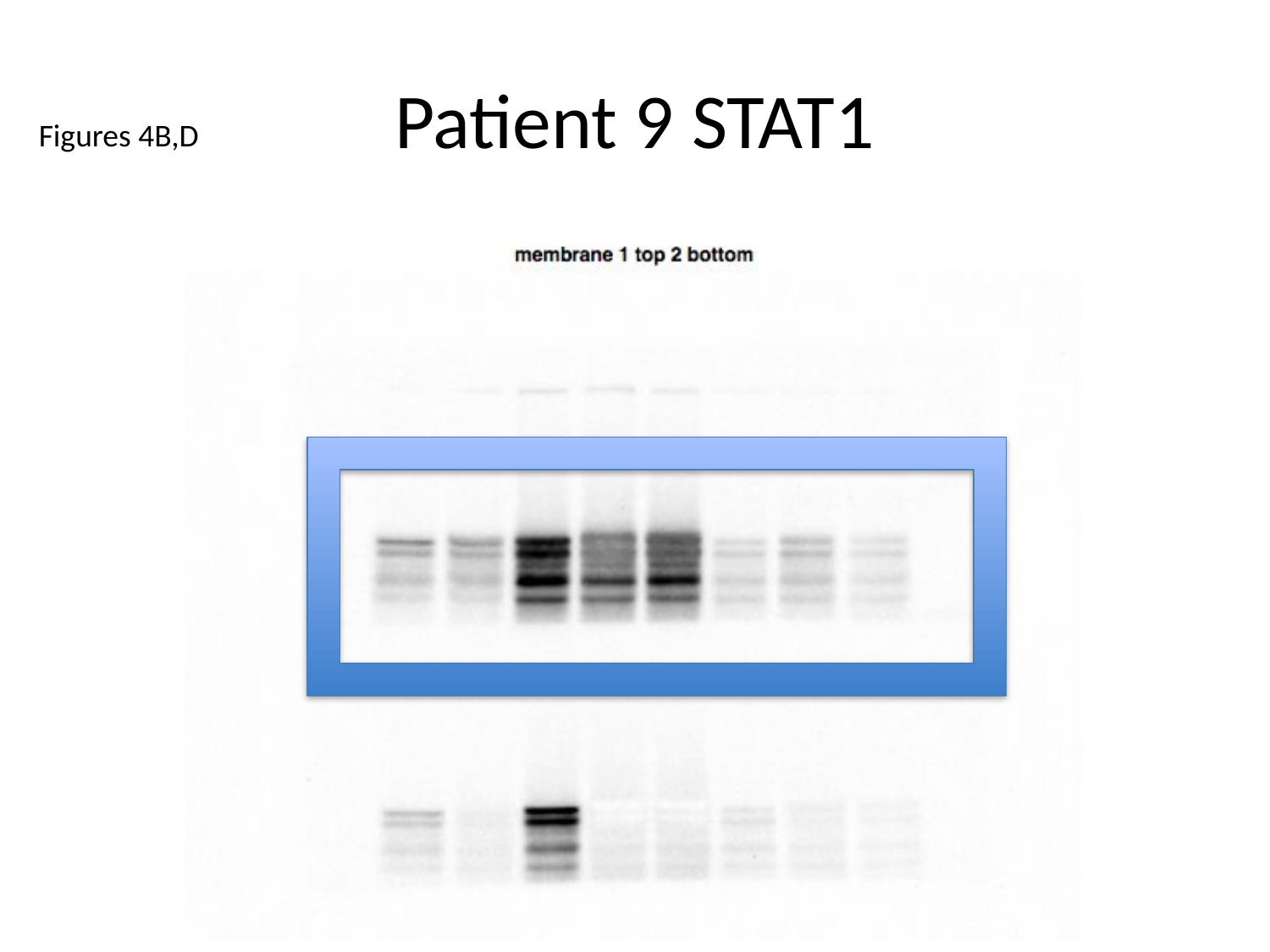

# Patient 9 STAT1
Figures 4B,D

## Slide 16
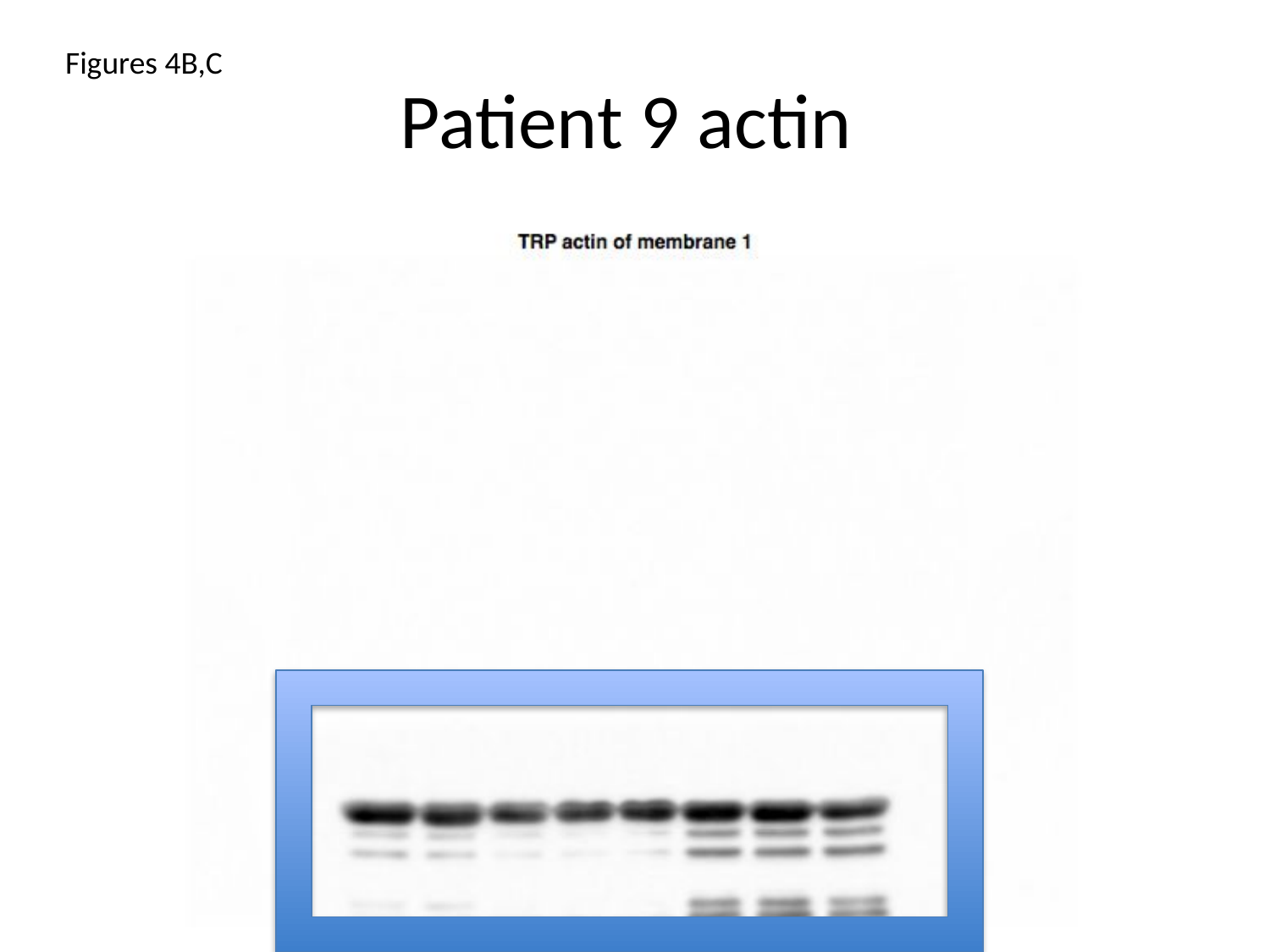

Figures 4B,C
# Patient 9 actin

## Slide 17
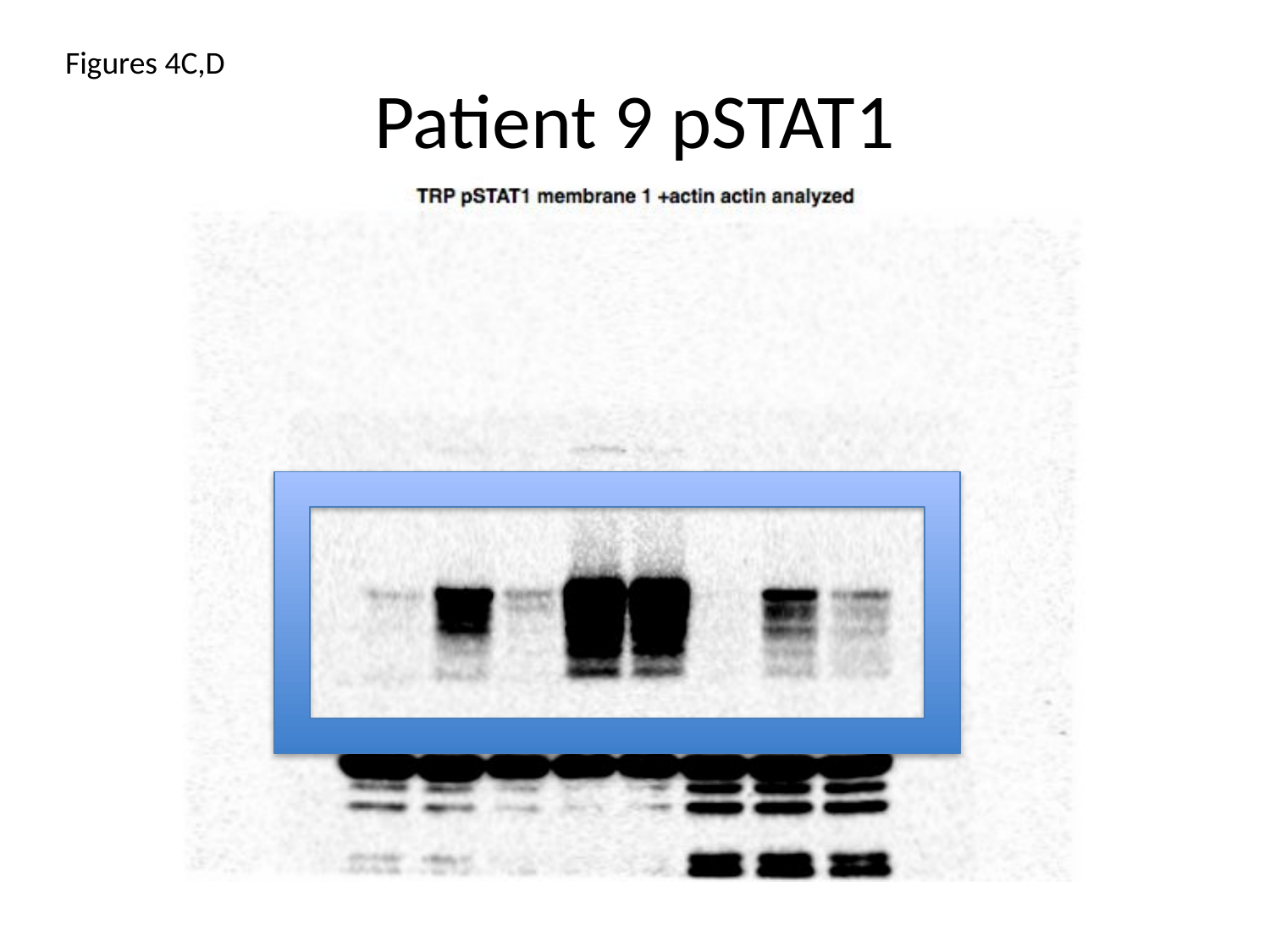

Figures 4C,D
# Patient 9 pSTAT1

## Slide 18
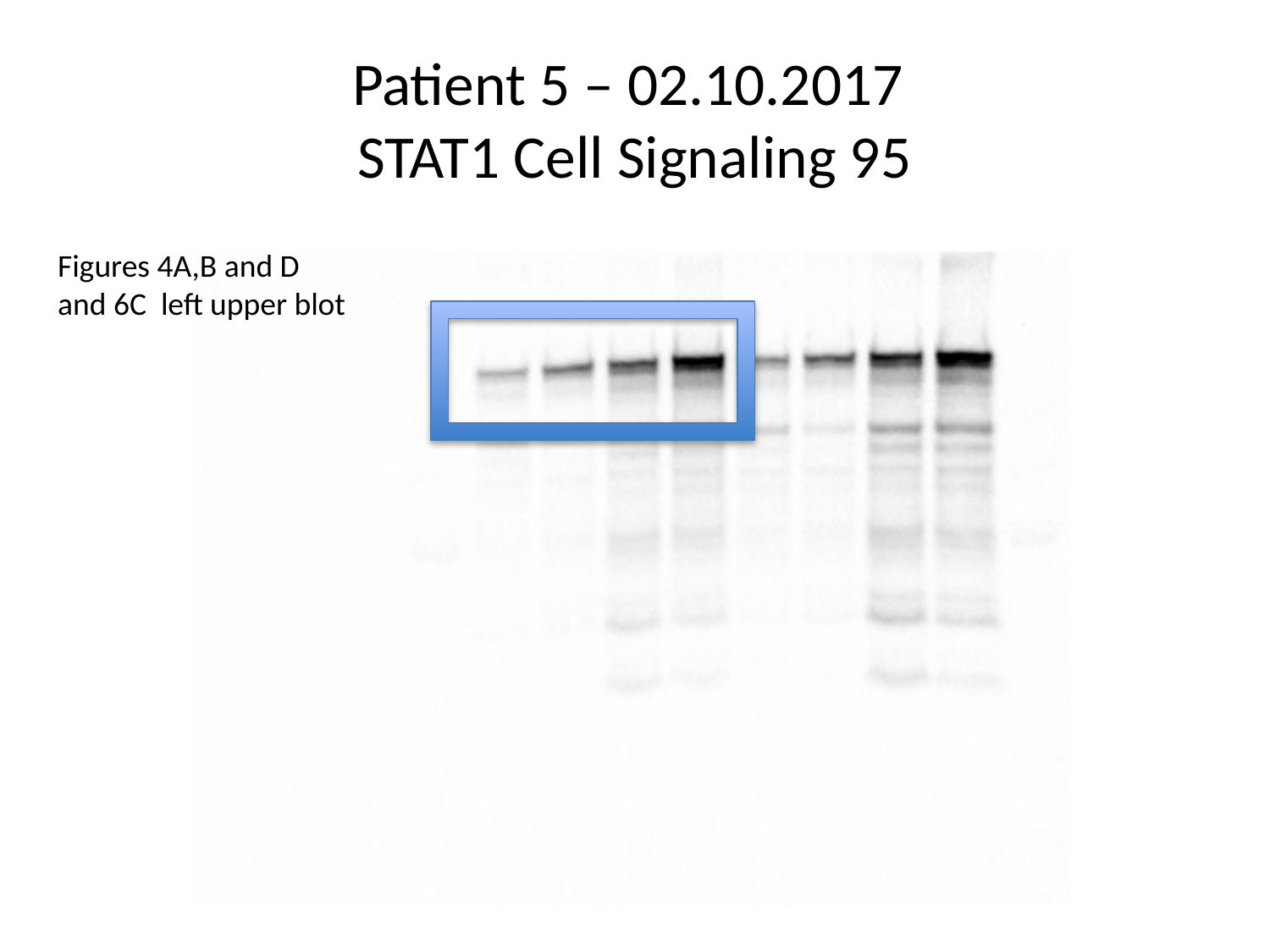

# Patient 5 – 02.10.2017 STAT1 Cell Signaling 95
Figures 4A,B and D
and 6C left upper blot

## Slide 19
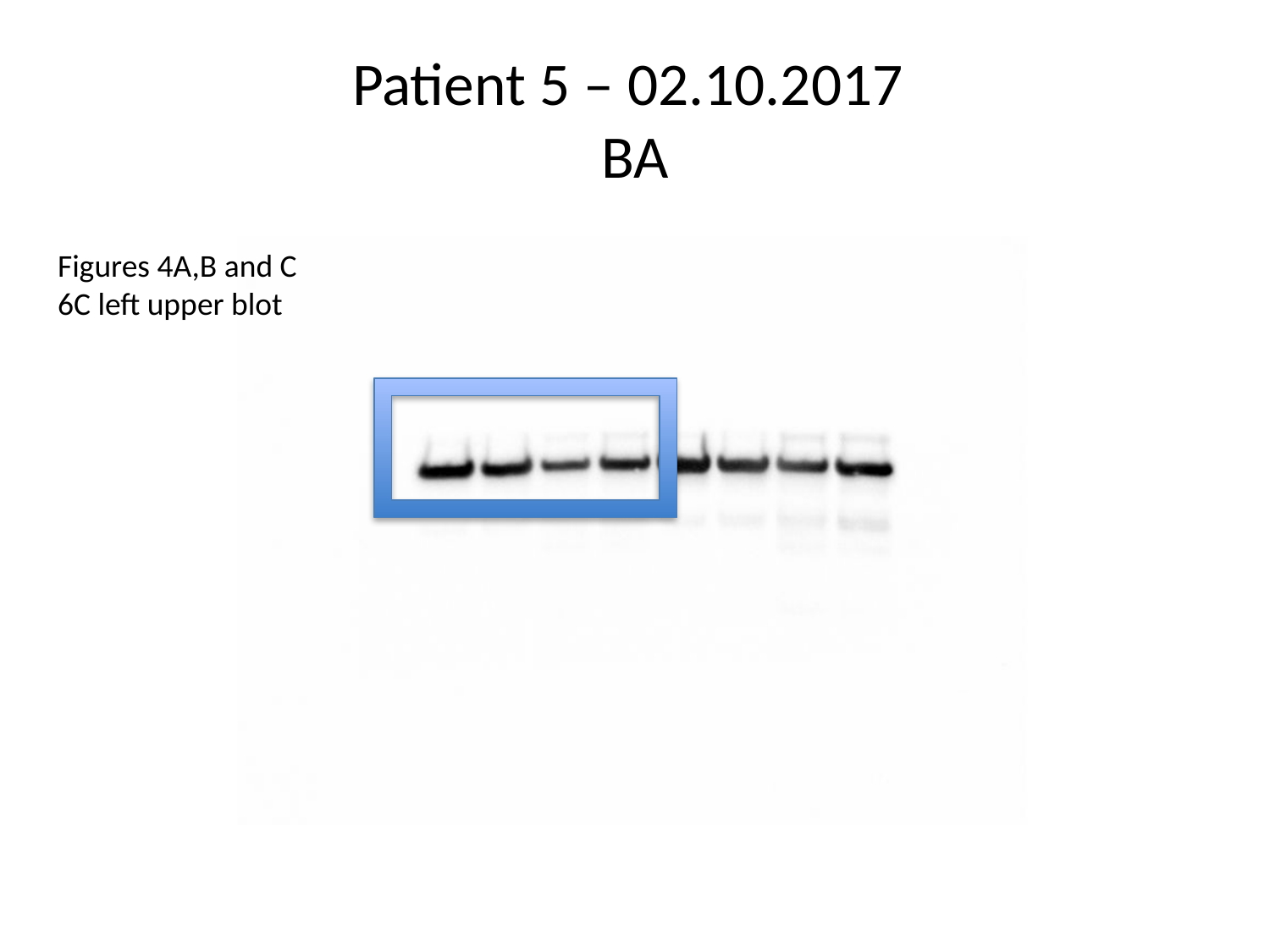

# Patient 5 – 02.10.2017 BA
Figures 4A,B and C
6C left upper blot

## Slide 20
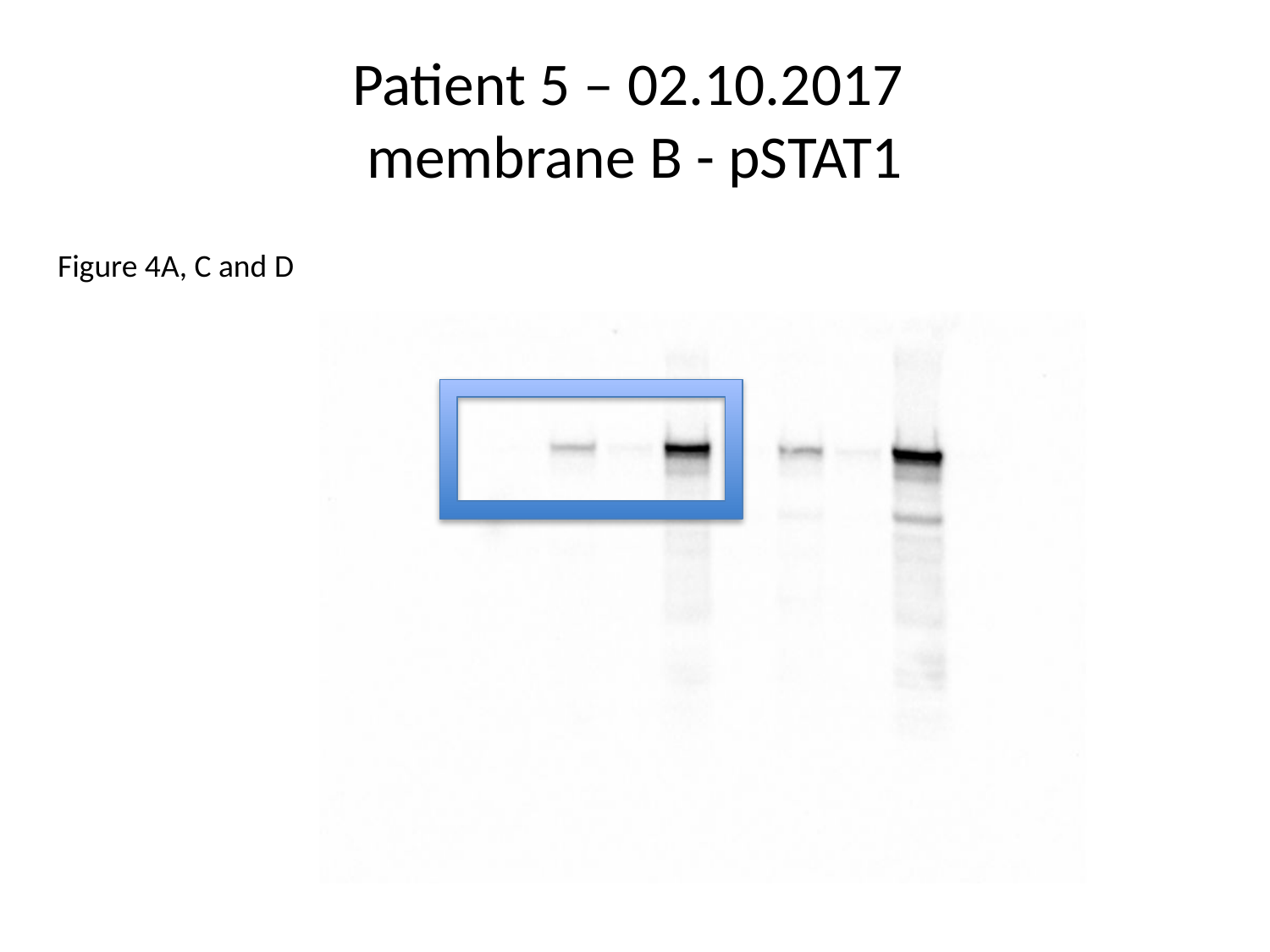

# Patient 5 – 02.10.2017 membrane B - pSTAT1
Figure 4A, C and D

## Slide 21
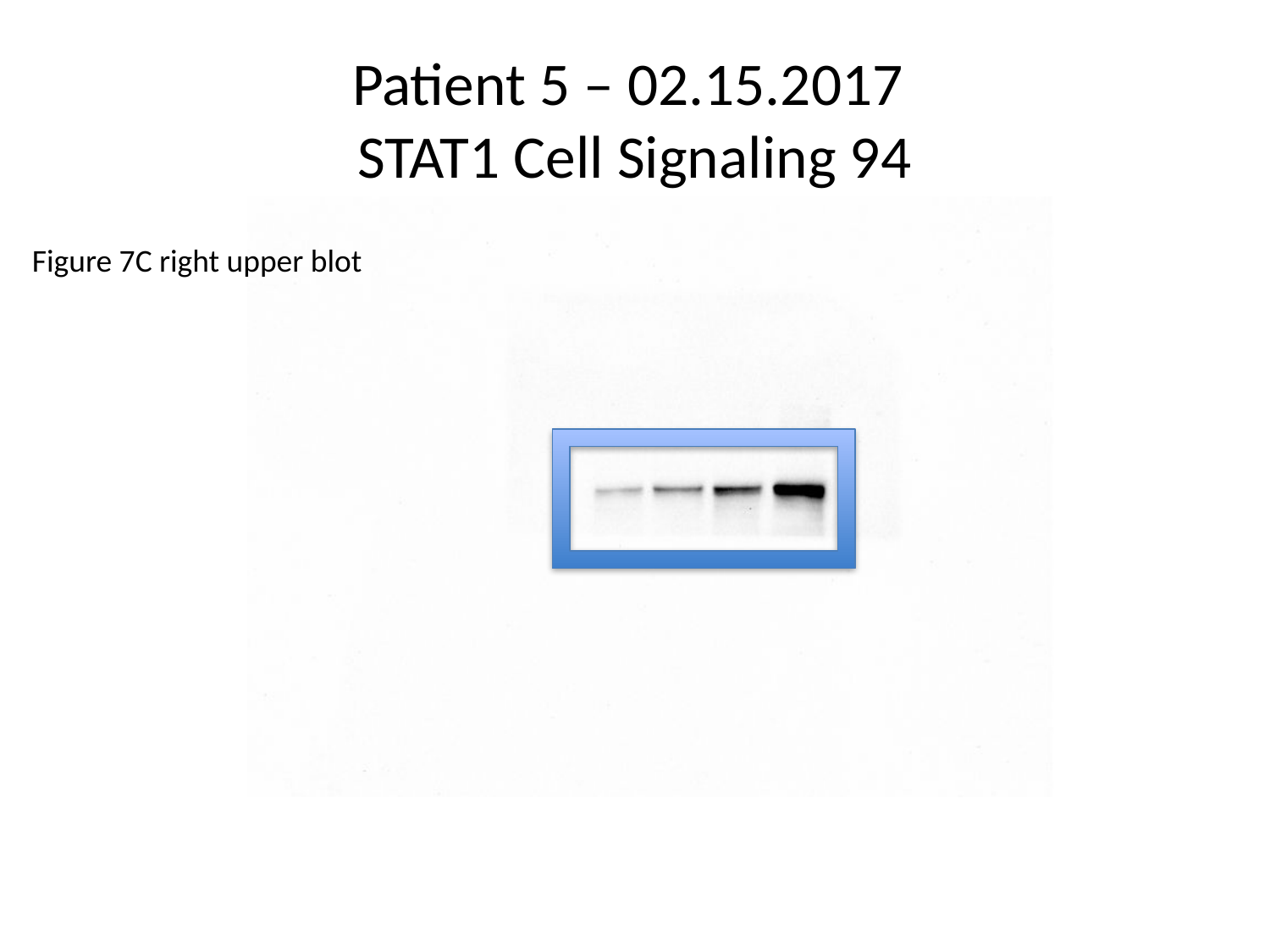

# Patient 5 – 02.15.2017 STAT1 Cell Signaling 94
Figure 7C right upper blot

## Slide 22
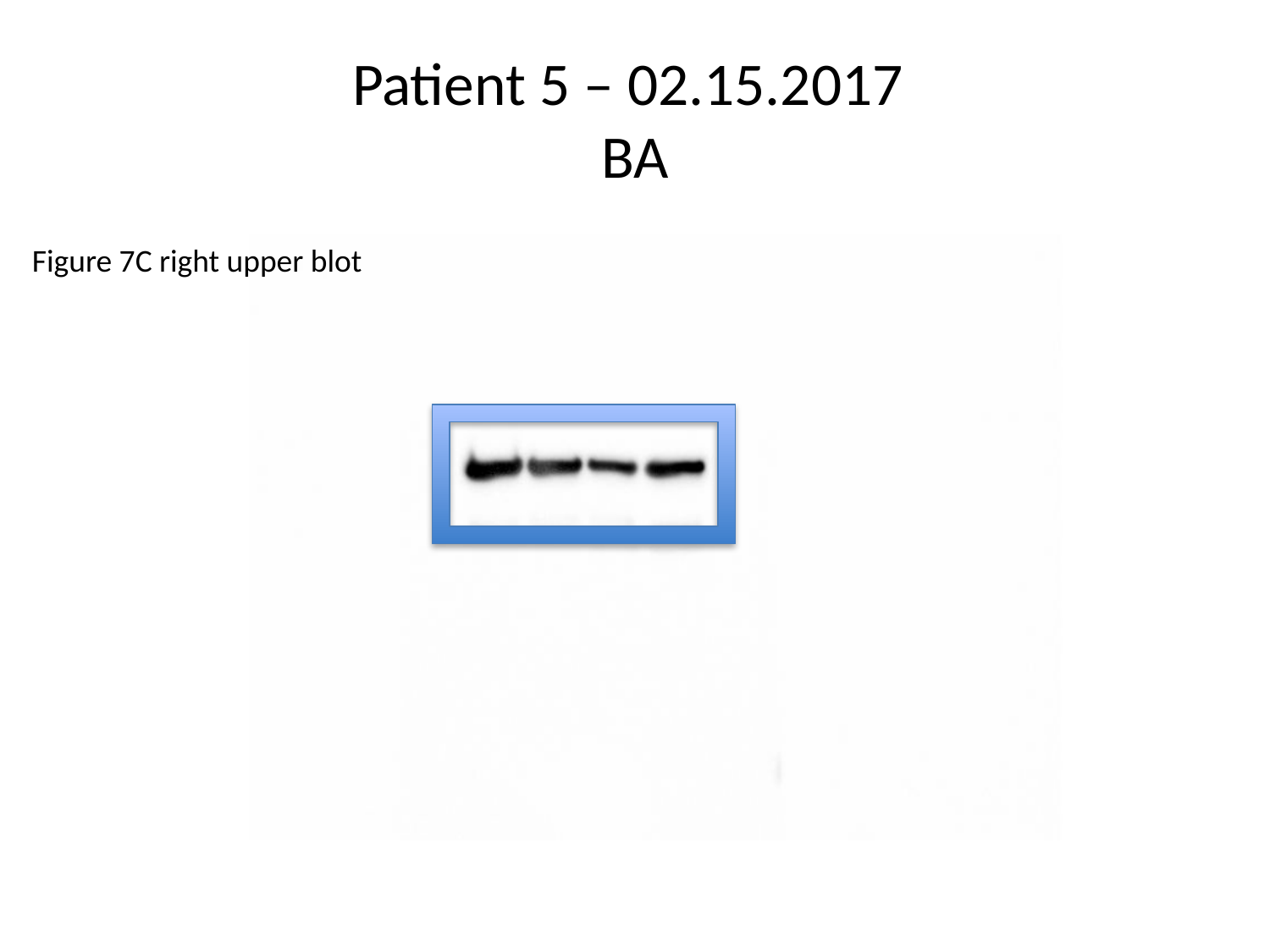

# Patient 5 – 02.15.2017 BA
Figure 7C right upper blot

## Slide 23
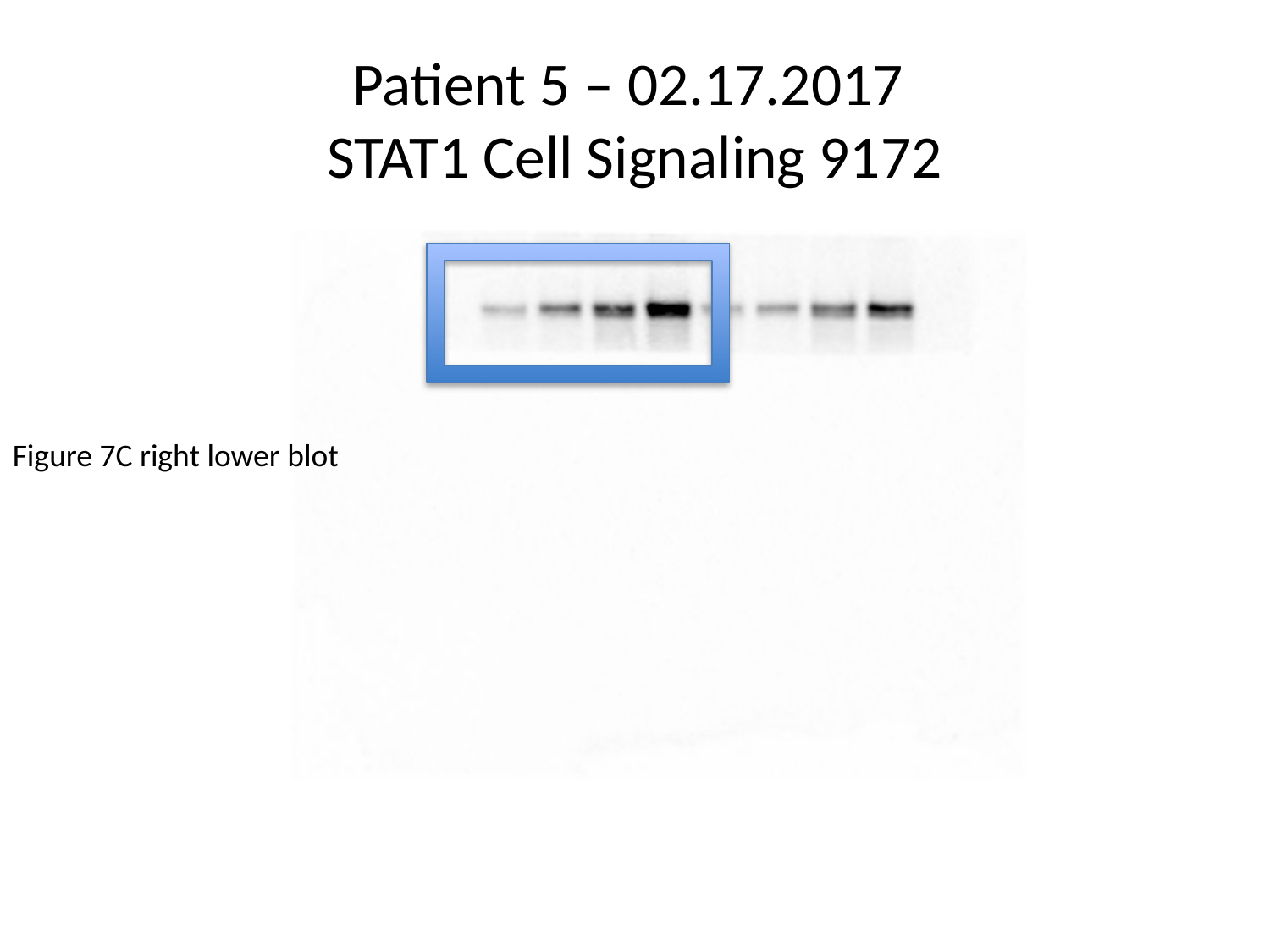

# Patient 5 – 02.17.2017 STAT1 Cell Signaling 9172
Figure 7C right lower blot

## Slide 24
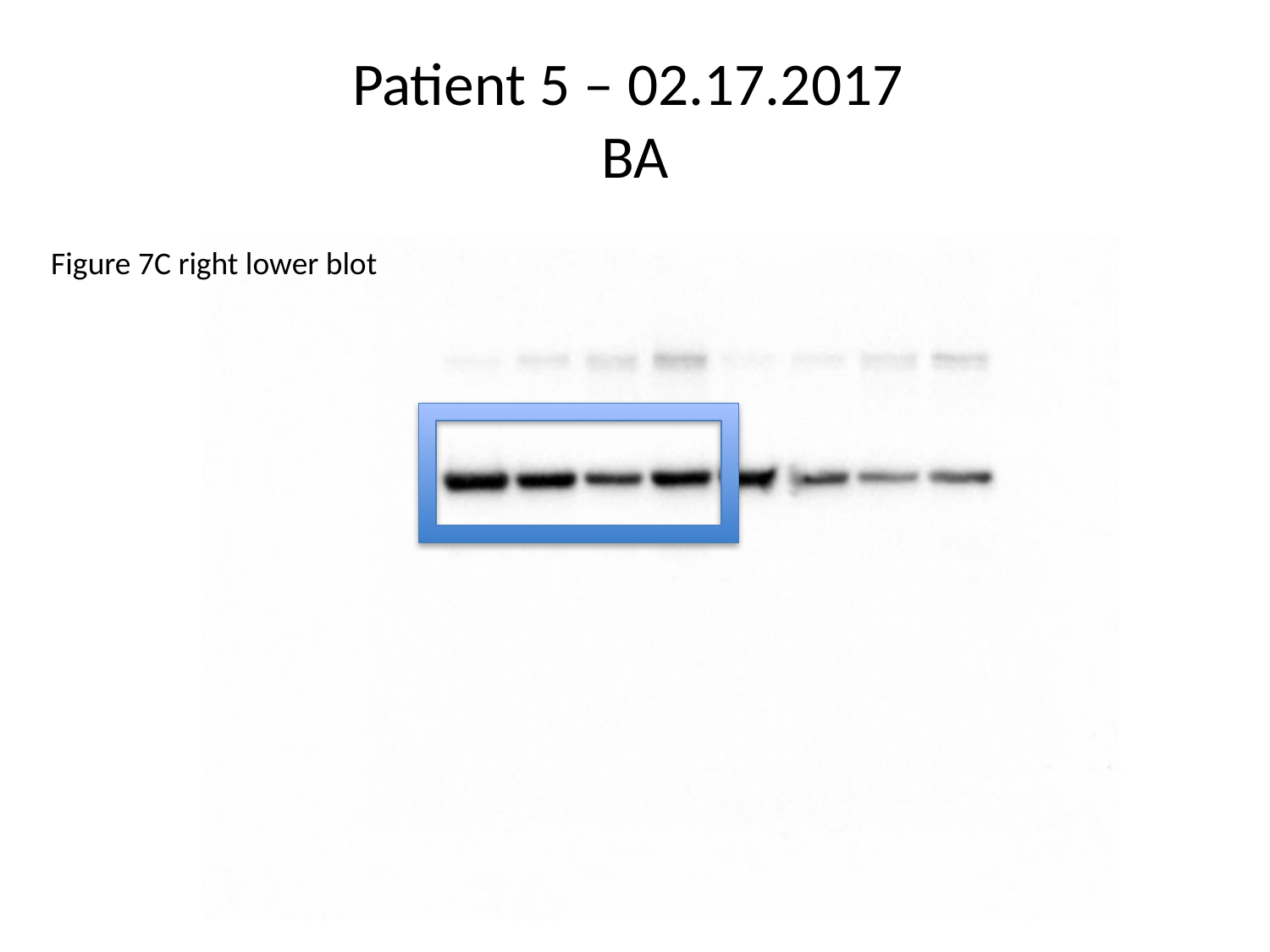

# Patient 5 – 02.17.2017 BA
Figure 7C right lower blot

## Slide 25
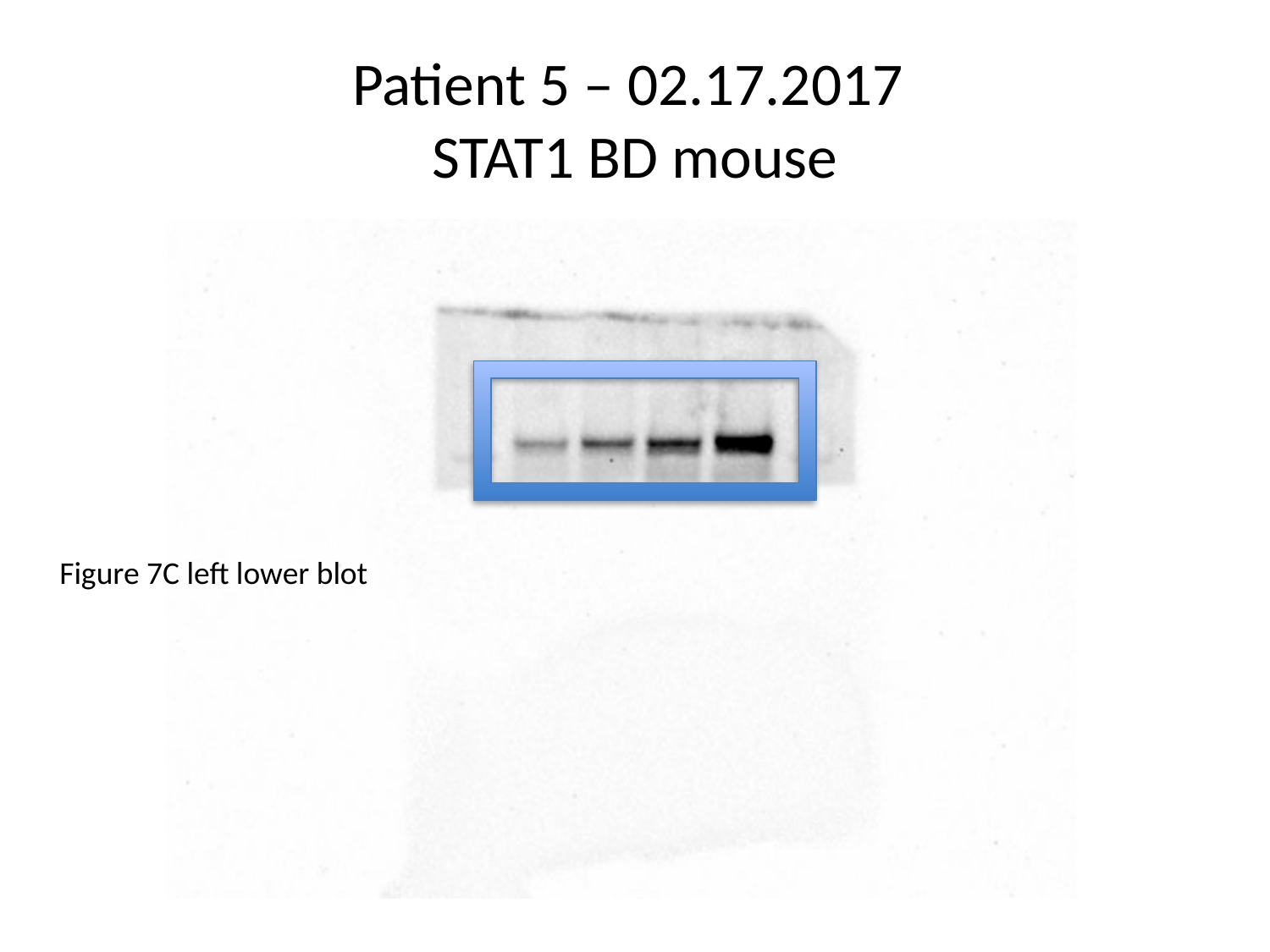

# Patient 5 – 02.17.2017 STAT1 BD mouse
Figure 7C left lower blot

## Slide 26
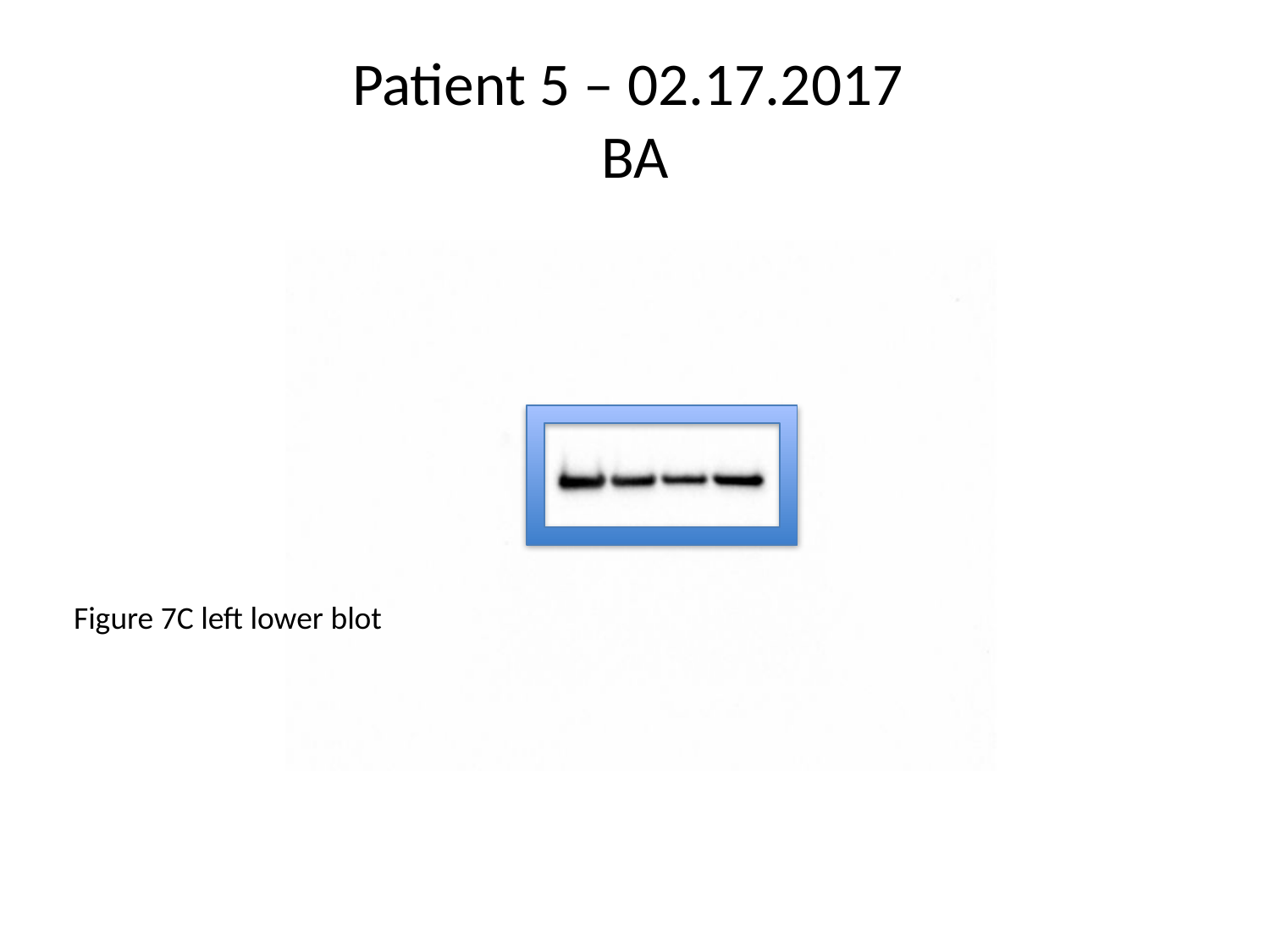

# Patient 5 – 02.17.2017 BA
Figure 7C left lower blot

## Slide 27
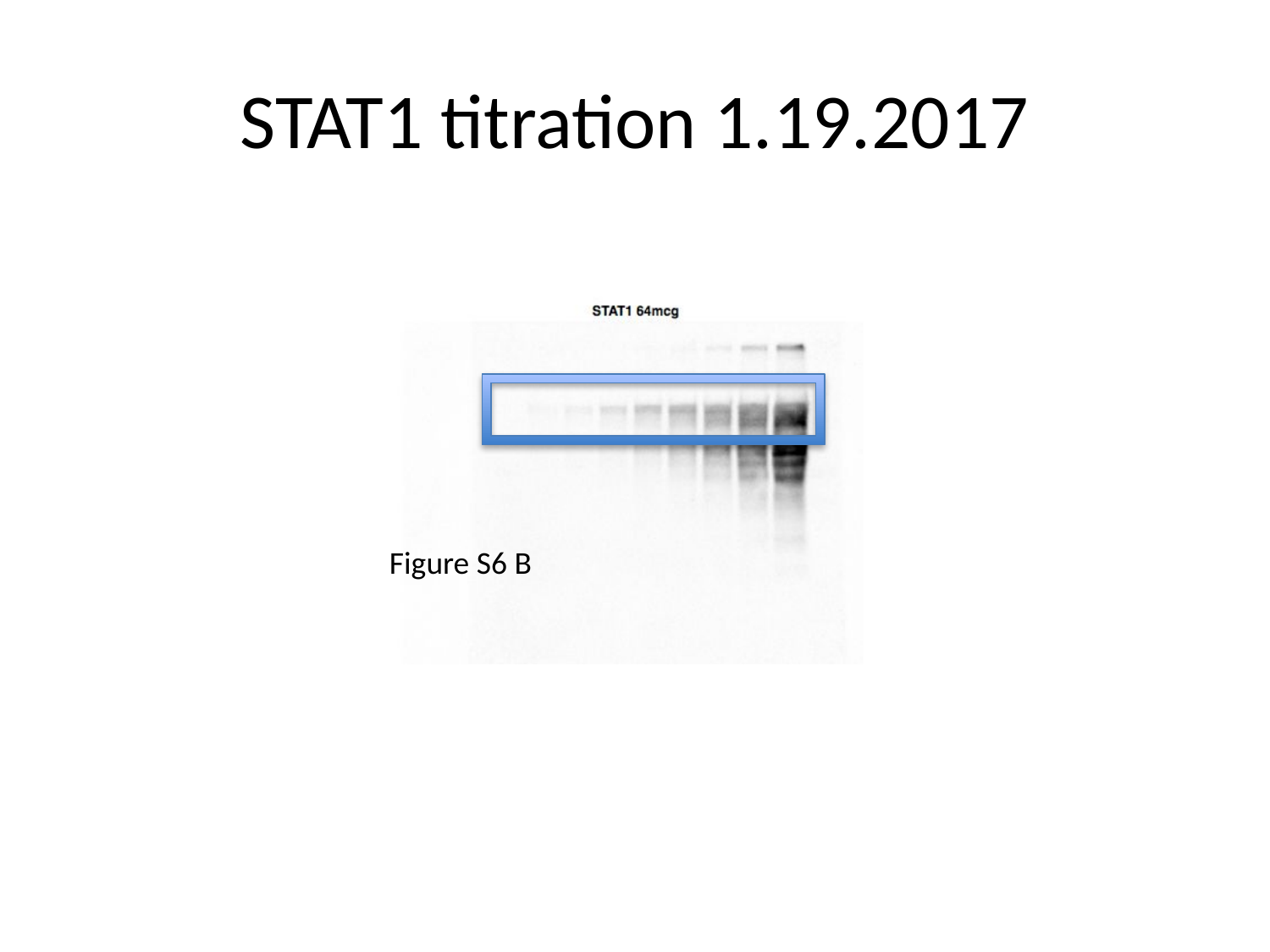

# STAT1 titration 1.19.2017
Figure S6 B

## Slide 28
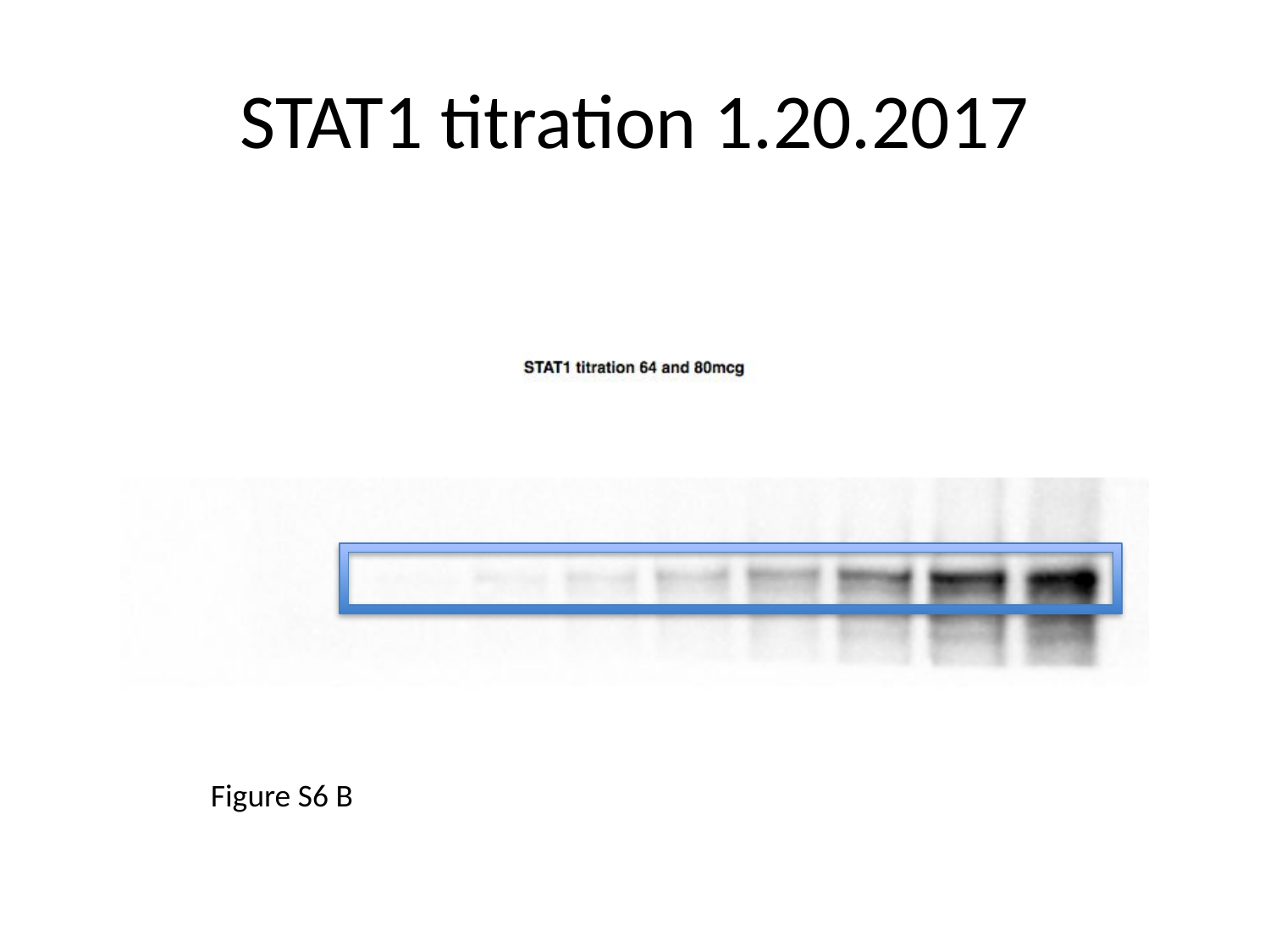

# STAT1 titration 1.20.2017
Figure S6 B

## Slide 29
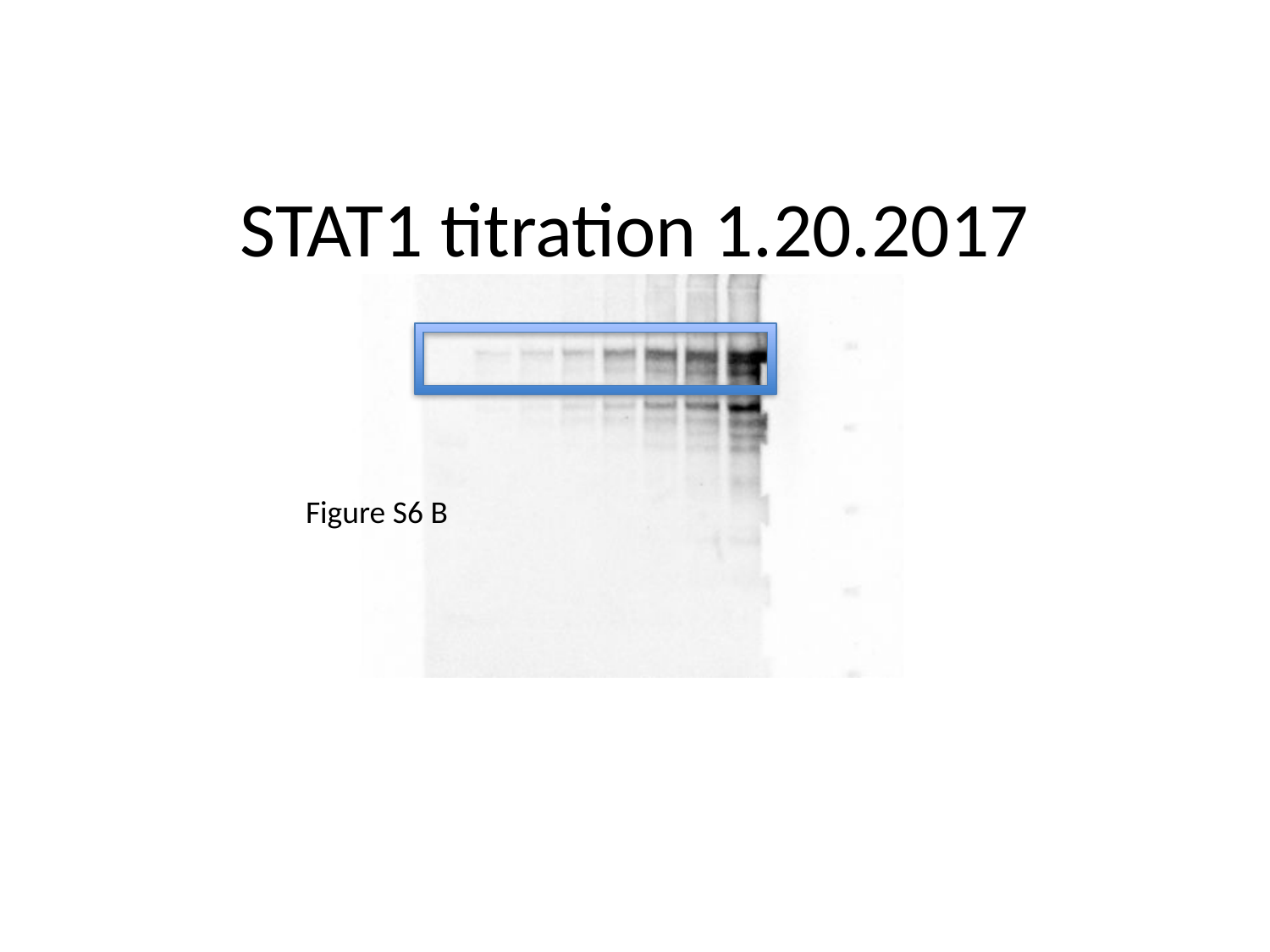

# STAT1 titration 1.20.2017
Figure S6 B

## Slide 30
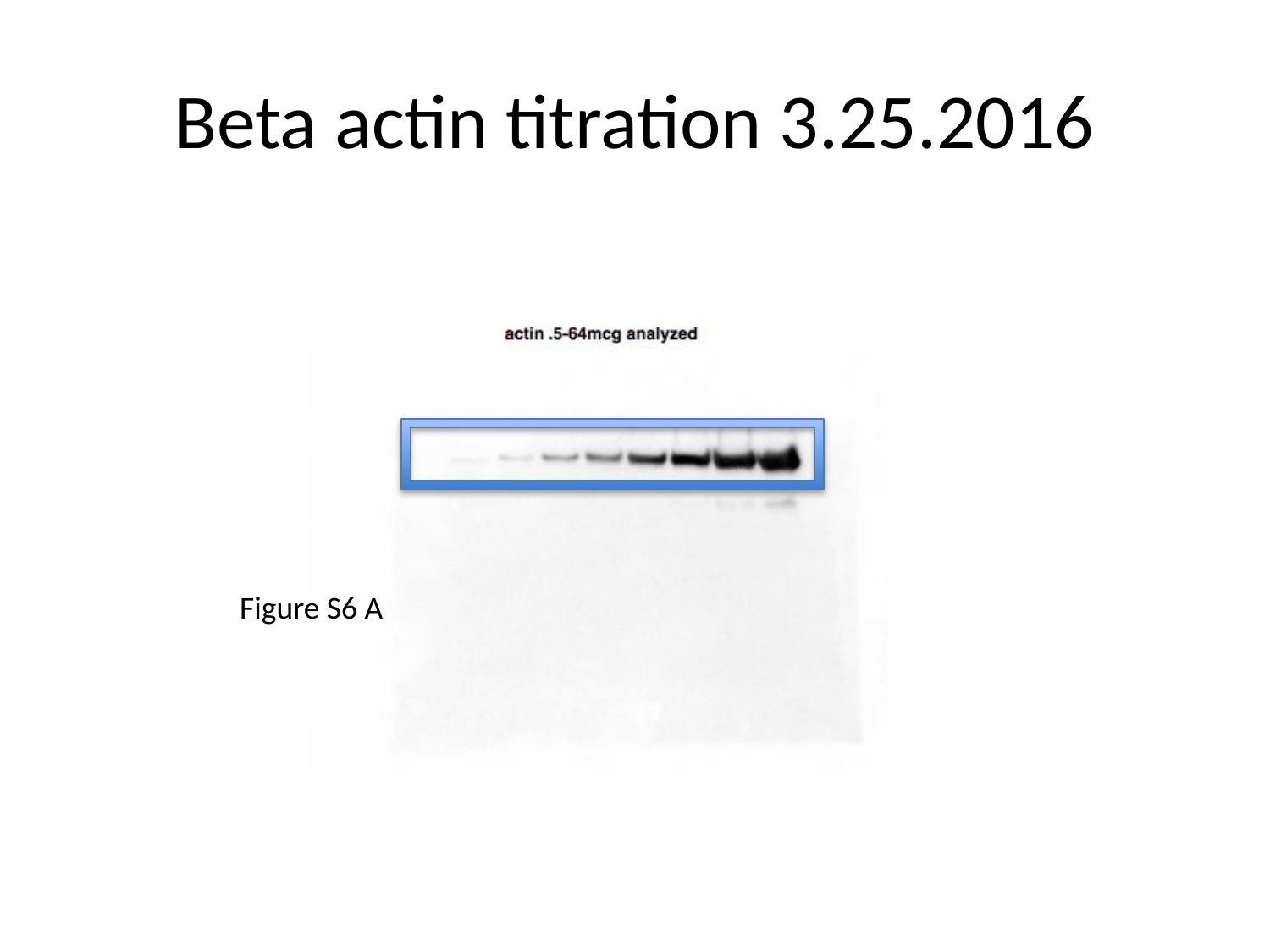

# Beta actin titration 3.25.2016
Figure S6 A
