# Supplementary figures and images for: STAT1 Gain-of-Function Mutations Cause High Total STAT1 Levels With Normal Dephosphorylation
Source: Front Immunol. 2019 Jul 10;10:1433. doi: 10.3389/fimmu.2019.01433 (PMC6635460; doi:10.3389/fimmu.2019.01433)

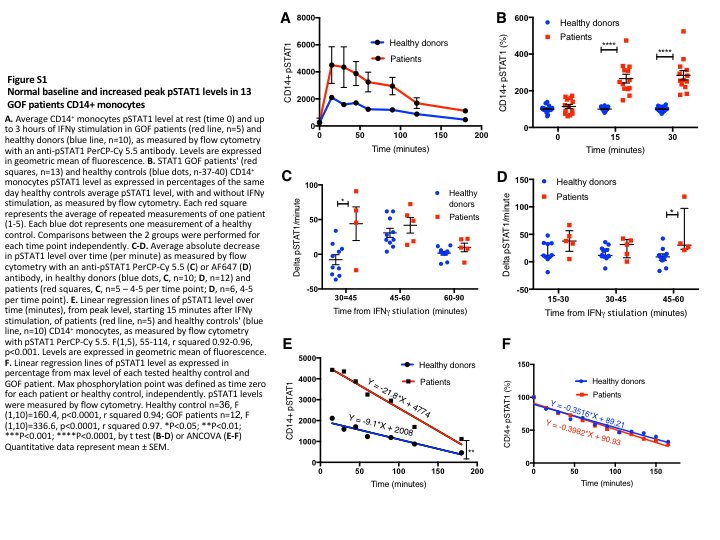

Supplement: Supplementary file 3 [file Image_1.tiff]

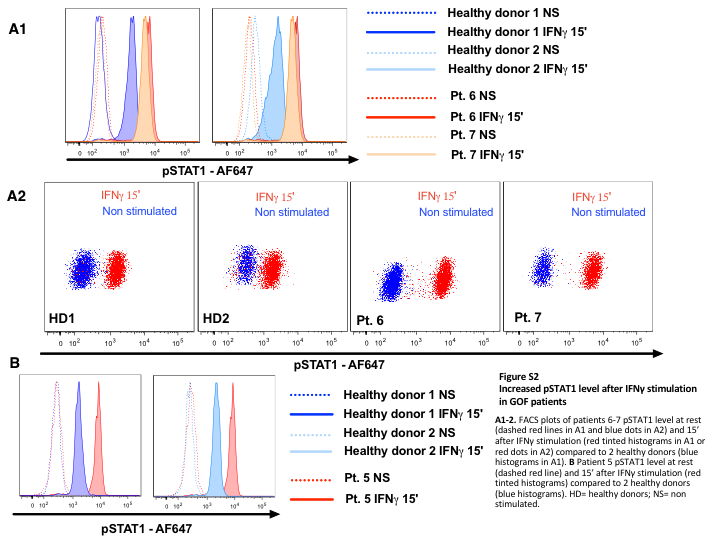

Supplement: Supplementary file 4 [file Image_2.tiff]

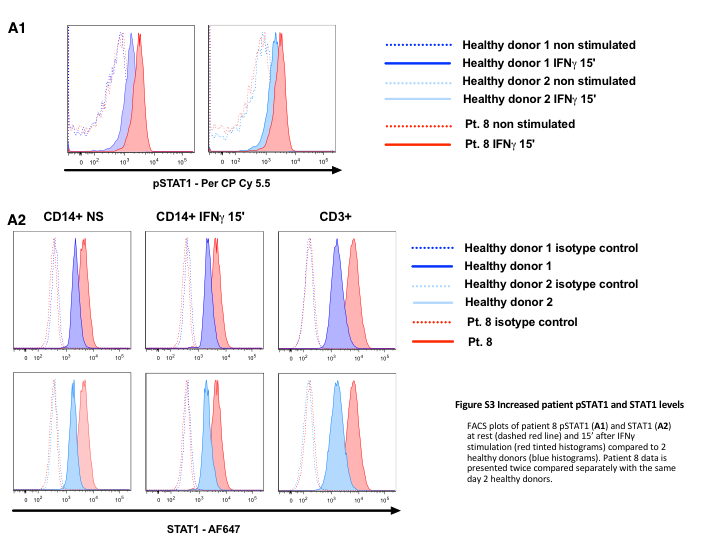

Supplement: Supplementary file 5 [file Image_3.tiff]

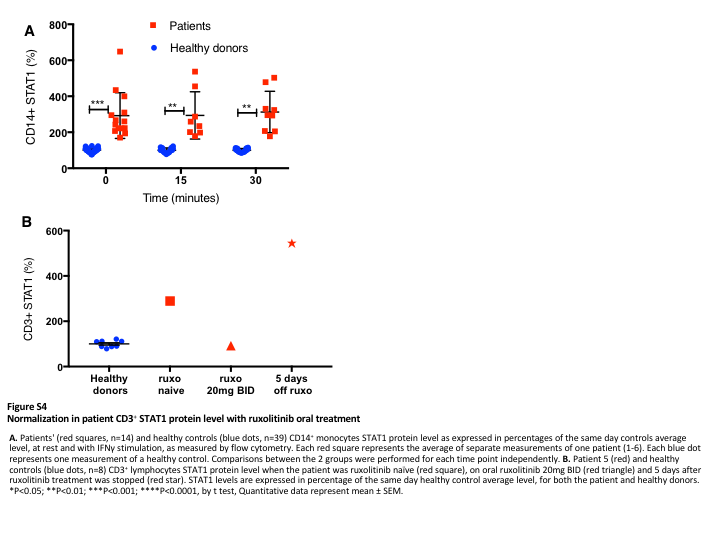

Supplement: Supplementary file 6 [file Image_4.tiff]

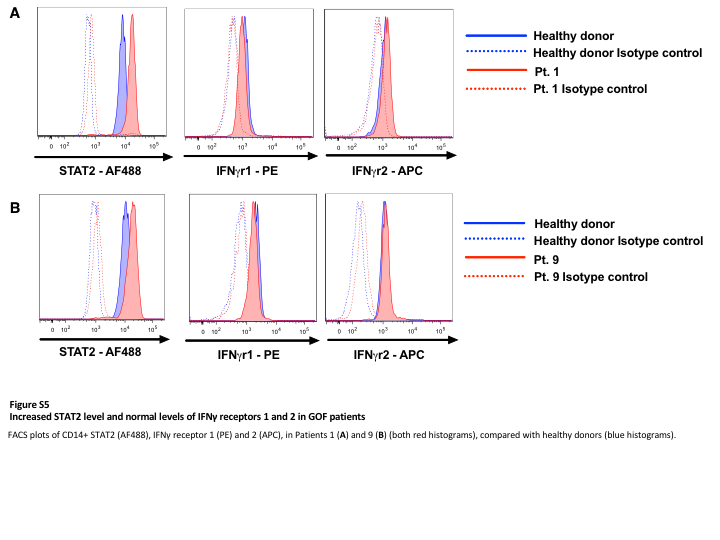

Supplement: Supplementary file 7 [file Image_5.tiff]

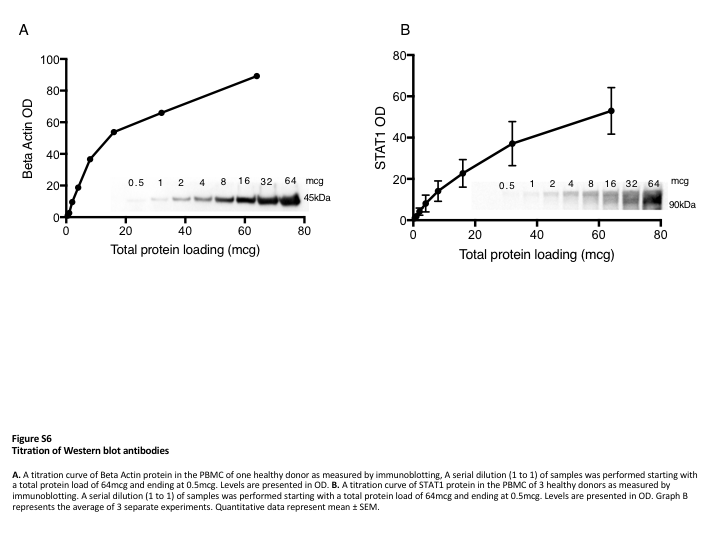

Supplement: Supplementary file 8 [file Image_6.tiff]
